# MicroRNA and mRNA Transcriptome Profiling in Primary Human Astrocytes Infected with *Borrelia burgdorferi*

**DOI:** 10.1371/journal.pone.0170961

**Published:** 2017-01-30

**Authors:** Timothy Casselli, Humaira Qureshi, Elizabeth Peterson, Danielle Perley, Emily Blake, Bradley Jokinen, Ata Abbas, Sergei Nechaev, John A. Watt, Archana Dhasarathy, Catherine A. Brissette

**Affiliations:** Department of Biomedical Sciences, University of North Dakota School of Medicine and Health Sciences, Grand Forks, ND, United States of America; University of Maryland, College Park, UNITED STATES

## Abstract

Lyme disease is caused by infection with the bacterium *Borrelia burgdorferi (Bb)*, which is transmitted to humans by deer ticks. The infection manifests usually as a rash and minor systemic symptoms; however, the bacteria can spread to other tissues, causing joint pain, carditis, and neurological symptoms. Lyme neuroborreliosis presents itself in several ways, such as Bell’s palsy, meningitis, and encephalitis. The molecular basis for neuroborreliosis is poorly understood. Analysis of the changes in the expression levels of messenger RNAs and non-coding RNAs, including microRNAs, following *Bb* infection could therefore provide vital information on the pathogenesis and clinical symptoms of neuroborreliosis. To this end, we used cultured primary human astrocytes, key responders to CNS infection and important components of the blood-brain barrier, as a model system to study RNA and microRNA changes in the CNS caused by *Bb*. Using whole transcriptome RNA-seq, we found significant changes in 38 microRNAs and 275 mRNAs at 24 and 48 hours following *Bb* infection. Several of the RNA changes affect pathways involved in immune response, development, chromatin assembly (including histones) and cell adhesion. Further, several of the microRNA predicted target mRNAs were also differentially regulated. Overall, our results indicate that exposure to *Bb* causes significant changes to the transcriptome and microRNA profile of astrocytes, which has implications in the pathogenesis, and hence potential treatment strategies to combat this disease.

## Introduction

Lyme disease (or Lyme borreliosis) is prevalent across the entire northern hemisphere, including Europe and parts of Asia [1]. In the United States, the Lyme disease spirochete, *Borrelia burgdorferi (Bb)*, is the cause of more than 90% of all arthropod-borne diseases affecting humans [2]. Roughly 30,000 cases are reported to the Centers for Disease Control and Prevention (CDC) every year, but the infection is likely underreported, and revised estimates suggest the rate is closer to 300,000 people affected by Lyme disease per year [1, 3, 4]. Total direct medical costs of Lyme disease and the controversial Post-Treatment Lyme Disease Syndrome (PTLDS) in the USA are estimated at $700 million- $1.3 billion per year [5].

Infectious *Bb* causes a multisystem disorder including neurological complications [6]. Neurological manifestations include cranial neuritis, facial nerve palsy, and meningitis [7–9]. More serious complications such as CNS vasculitis and hemorrhagic stroke, although rare, can occur [10–12]. As many as ten percent of antibiotic-treated patients may continue to suffer from post-treatment Lyme disease syndrome (PTLDS) [13], a disorder characterized by musculoskeletal pain, fatigue and cognitive complaints that persist for at least 6 months after treatment [14]. The pathophysiology behind the neurocognitive complaints of Lyme disease is unclear, but the inflammatory response to the bacterium or its components is likely to play a role [15, 16]. For example, patients with a history of Lyme disease and objective memory impairment have elevated serum IFN-α levels compared to healthy controls, which remain elevated despite antibiotic treatment [17], and human glia stimulated with *Bb* in vitro suggest that the inflammatory milieu directly contributes to apoptosis of neurons [18–20].

There have been limited transcriptome analyses of human cells in culture in response to *Bb* [21–23]. To build on these limited data sets, we chose to focus on transcriptional profiling of gene and microRNA expression changes. MicroRNAs are small noncoding RNAs involved in post-transcriptional regulation of gene expression through RNA silencing, mainly by binding to the 3’ untranslated region of a target mRNA [24]. We chose to profile transcriptional responses to *Bb* in astrocytes, abundant cells in the central nervous system that provide nutrients, recycle neurotransmitters, and maintain homeostasis [25]. Astrocytes directly play active roles in the transfer and storage of information in the brain, and the coordinated action of both neurons and astrocytes are necessary to maintain synaptic plasticity [26]. Astrocytes are also key responders to CNS injury and infection, responses that must be balanced to eliminate threats while preserving surrounding tissue and without causing neurological impairment [27]. We have previously shown robust chemokine expression from astrocytes stimulated with *Bb*, including attractants for monocytes, neutrophils, and T cells [28]. If uncontrolled in the context of neuroborreliosis, the astrocyte response could lead to long-term injury in the CNS.

Using primary astrocytes in culture, we demonstrate differential expression of over 200 genes following infection with *Bb*, as well as changes in 38 microRNAs following 48 hrs of infection. Pathway analysis of transcriptional changes revealed gene categories that included developmental pathways, chromatin assembly, cell-cell adhesion, and immune system processes. A subset of transcription factors as well as long non-coding RNAs also change in expression, suggesting that regulatory networks could be altered following the infection, resulting in long-term changes to the transcriptome. The microRNA profiling revealed changes in expression of microRNAs involved in cell adhesion and several signaling pathways. Additionally, over half of genes shown to be differentially expressed during co-culture with *Bb* were predicted to act as targets for one or more of the miRNAs that were concurrently differentially expressed. Taken together, we present for the first time, a catalog of differential gene and microRNA expression changes in astrocytes following *Bb* infection.

## Results

### RNA-seq reveals changes in the astrocyte transcriptome following *Bb* infection

**Fig 1:**
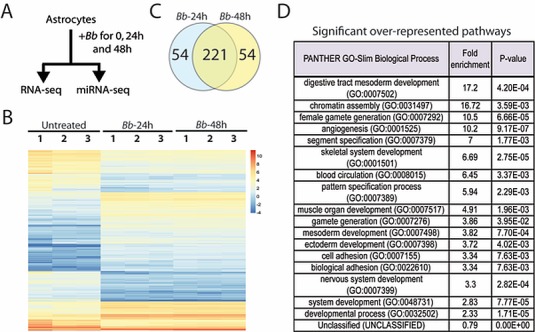
Transcriptome changes in astrocytes following *Bb* infection. Primary human astrocytes were cultured untreated or treated with *Bb* for 24h (N = 3) or 48h (N = 3) respectively, and total RNA and microRNAs isolated from the same preparation (A) and subject to massively parallel sequencing (see text for details). The results were plotted as a heat map to reveal significant differences in gene expression (B; 1, 2, 3 represent individual biological replicates), with the largest number of gene expression changes being seen at the 48h time point (C). PANTHER lysis (D) revealed a number of developmental and cell-adhesion pathways were affected.

**Fig 2:**
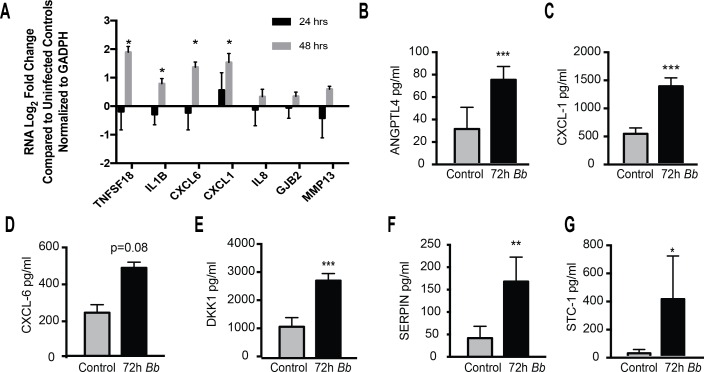
Validation of RNA-sequencing results. (A) Gene expression changes seen in a subset of genes following *Bb* treatment were validated. cDNA was prepared and amplified using primers specific for TNFSF18, IL1beta, CXCL1, CXCL6, GJB2, MMP13, and the housekeeping gene GAPDH. Data are expressed as the ratio of gene expression (Log_2_FC) in *Bb*-treated cells compared to untreated controls (n = 3) for each gene of interest normalized against GAPDH expression calculated using the delta-delta Ct method [29]. Asterisks indicate significant difference compared to uninfected controls as determined by one-way ANOVA followed by Holm-Sidak (p<0.01). (B-G) ELISAs were performed using control and astrocytes treated with *Bb* for 72h. Data represent the mean and standard errors of the concentration of cytokine in the supernatant from two independent biological replicates analyzed in triplicate for each condition. Asterisks indicate significant differences as determined using Student’s t-test.

To date, there are limited studies examining the transcriptome changes in any cell type co-cultured with *Bb*. We performed a time course of *Bb* infection in primary human astrocytes in culture (3 biological replicates per time point) and examined genome-wide RNA changes relative to untreated cells at 24 and 48 hours after infection (Fig 1A). At each time point, we isolated RNA and microRNA, created Illumina sequencing libraries, and then sequenced each time point using next-generation sequencing on Illumina’s HiSeq2000 (RNA) and the MiSeq (microRNA) as described in the materials and methods. Multidimensional scaling (MDS) plots (S1 Fig) indicated that the replicates clustered together with no outliers, and there were clear differences between the untreated and *Bb* treatments for each day, also noted by heat map analyses (Fig 1B). After data normalization, we extracted only those genes with a four-fold (log_2_FC = 2) change or higher in expression (up or down) and an adjusted p-value (false discovery rate or FDR) of 0.05 or lower. Using this filtered set of genes, we observed alterations in steady state levels of 275 transcripts following 24 or 48 hrs of *Bb* infection, with considerable overlap among differentially expressed genes at both timepoints (Fig 1C and S1 File). A previous study utilized microarray analysis to examine genes differentially expressed in Rhesus macaque primary microglia after co-culture with *Bb* [15]. Of the 275 genes we found to be differentially expressed in the current study, 43 of those (16%) were reported by Myers et al as being differentially expressed in the same direction (i.e. up or down) in Rhesus macaque microglia after co-culture with *Bb*. A subset of differentially expressed genes identified by RNA-seq were further validated by RT-PCR following exposure to *Bb* relative to untreated astrocytes. In agreement with our RNA-seq dataset, the highly upregulated genes TNSF18, IL1B, CXCL6, and CXCL1 were also shown by RT-PCR to be significantly upregulated after 48 hours co-incubation with *Bb* (Fig 2A).

We categorized the sets of genes that changed following *Bb* infection for each time point using the PANTHER classification system (pantherdb.org) [30]. Genes involved in development, chromatin assembly, cell-cell adhesion and angiogenesis were among the top pathways identified as changing significantly in expression following *Bb* infection (Fig 1D). Next, network analysis using Ingenuity Pathway Analysis (IPA) tools revealed changes in networks at 24 hours (S2 and S3 Files), including those involving metabolism (S2 and S3 Files, network1), development and disease (S2 and S3 Files, networks 2 and 3), and connective tissue disorders (S2 and S3 Files, network 4). Changes in networks at 48 hours (S2 and S3 Files) included those involving cancer and connective tissue disorders (S2 and S3 Files, network 1), neurological disease (S2 and S3 Files, network 2), cell-cell signaling (S2 and S3 Files, network 3) and cellular movement and immune cell trafficking (S2 and S3 Files, network 9).

Several genes involved in inflammation and immune response, including those previously implicated in Lyme disease, such as *il1b* and *cxcl8*, were differentially regulated at 24 and 48 hours (Table 1) [16, 28, 31–33]. Further, several transcription factors (Table 2), including Forkhead box (FOX) protein family members were upregulated, while other regulators of transcription such as the homeobox (*HOX*) genes such as engrailed homeobox 2, and several *HOX* cluster genes were downregulated in response to *Bb*. *HOX* cluster genes appear to be among the most strongly differentially expressed gene sets (Table 2), which have not previously been implicated in *Bb* pathogenesis. Homeobox genes are highly conserved transcription factors which are involved in development and body patterning [34]. Forkhead box (FOX) proteins are a family of transcriptional regulatory proteins that control diverse cellular processes including differentiation, metabolism, development, proliferation, and apoptosis [35–38].

**Table 1:** Selected inflammation and immune function genes altered in response to *Bb*.

		24h		48h	
Gene symbol	Gene name	FC	p-Value	FC	p-Value
TNFSF18	Tumor necrosis factor (ligand) superfamily, member 18	439.59	6.85E-37	580.04	5.04E-45
IL1B	Interleukin-1 beta	42.81	1.02E-36	36.50	7.16E-33
TXK	TXK tyrosine kinase	18.25	3.68E-21	31.34	5.45E-33
ERAP2	Endoplasmic reticulum aminopeptidase 2	19.43	1.97E-28	23.10	2.04E-31
CXCL6	Chemokine (C-X-C motif) ligand 6	18.13	4.42E-77	22.16	4.89E-86
CHI3L1	Chitinase 3-like 1 (cartilage glycoprotein-39)	26.17	3.78E-41	18.00	6.05E-30
CXCL8	Interleukin-8	18.77	8.32E-11	16.68	3.27E-10
DPP4	Dipeptidyl-peptidase 4	12.55	9.17E-43	13.83	9.66E-46
SRGN	Serglycin	6.32	4.17E-61	7.62	5.50E-73
CXCL1	Chemokine (C-X-C motif) ligand 1	5.28	4.93E-13	6.63	2.95E-16
EBI3	Epstein-Barr virus induced 3	4.41	9.42E-13	5.58	1.09E-17
NFATC2	Nuclear Factor of Activated T-Cells 2	-3.61	2.48E-06	-4.38	8.98E-08
CSF1R	Colony Stimulating Factor 1 Receptor	-3.12	2.37E-13	-5.06	2.23E-21
CD83	CD83 Molecule	-6.36	1.19E-07	-5.24	1.75E-06
HLA-DPB1	Major Histocompatibility Complex, Class II, DP Beta 1	-5.31	1.41E-13	-5.54	5.37E-14
CMKLR1	Chemerin Chemokine-Like Receptor 1	-1.36	0.04	-5.66	4.32E-20
ICOSLG	Inducible T-Cell Costimulator Ligand	-4.44	5.05E-16	-6.02	2.94E-19
CX3CL1	C-X3-C Motif Chemokine Ligand 1	-4.76	2.08E-13	-6.19	9.08E-17
HLA-DPA1	Major Histocompatibility Complex, Class II, DP Alpha 1	-11.08	3.70E-66	-7.94	2.65E-53
LSP1	Lymphocyte-Specific Protein 1	-12.21	8.84E-84	-9.38	2.15E-71
IL21R	Interleukin 21 Receptor	-16.45	1.22E-10	-12.91	2.01E-09
RARRES2	Retinoic Acid Receptor Responder 2	-22.94	6.48E-11	-13.83	1.22E-08

**Table 2:** Selected transcription factors altered in response to *Bb*.

		24h		48h	
Gene symbol	Gene name	FC	p-Value	FC	p-Value
EN2	engrailed homeobox 2	-16.02	6.07E-30	-27.72	2.10E-34
ERG	v-ets avian erythroblastosis virus E26 oncogene homolog	-15.40	4.26E-11	-10.26	6.06E-09
FOSB	FBJ murine osteosarcoma viral oncogene homolog B	-6.27	6.84E-06	-5.14	4.78E-05
FOXA1	forkhead box A1	4.63	1.86E-08	6.73	2.11E-12
FOXF1	forkhead box F1	4.29	6.94E-22	5.69	2.04E-30
FOXF2	forkhead box F2	5.27	1.51E-32	5.97	9.40E-38
FOXL2	forkhead box L2	5.89	1.85E-19	6.53	1.91E-21
FOXQ1	forkhead box Q1	18.35	4.32E-23	12.21	4.88E-16
HOXA2	homeobox A2	-22.00	1.49E-55	-14.14	1.20E-46
HOXA3	homeobox A3	-19.36	3.76E-74	-14.92	3.54E-65
HOXA4	homeobox A4	-20.61	4.62E-57	-11.97	2.89E-45
HOXA5	homeobox A5	-19.10	1.18E-43	-11.66	1.61E-35
HOXB3	homeobox B3	-4.26	2.80E-65	-4.14	7.56E-63
HOXB4	homeobox B4	-5.05	1.25E-39	-4.94	1.33E-38
HOXC4	homeobox C4	-12.83	8.31E-38	-8.25	3.35E-30
HOXD4	homeobox D4	-29.00	1.23E-32	-45.37	8.63E-34
MYOCD	myocardin	7.14	1.74E-13	8.62	7.46E-16
NKX2-4	NK2 homeobox 4	25.76	5.95E-35	30.13	3.89E-40
NUPR1	nuclear protein, transcriptional regulator, 1	-6.92	1.10E-05	-4.32	6.28E-04
OSR1	odd-skipped related transcription factor 1	4.05	1.41E-21	4.03	2.94E-21
PAX3	paired box 3	-7.85	6.93E-84	-7.20	8.25E-78
RCOR2	REST corepressor 2	-4.81	1.08E-26	-6.81	1.56E-35
SHOX2	short stature homeobox 2	60.87	2.61E-64	51.93	9.97E-58
SIX3	SIX homeobox 3	5.88	3.94E-53	6.24	2.14E-56
SIX6	SIX homeobox 6	78.76	8.98E-117	113.25	1.40E-147
TBX18	T-box 18	4.93	7.91E-53	4.18	6.68E-43
TCF21	transcription factor 21	7.80	6.30E-24	9.37	3.45E-29
TFCP2L1	transcription factor CP2-like 1	-4.07	5.49E-54	-4.50	1.05E-59

In addition to mRNA expression changes in response to *Bb*, a number of non-coding RNAs were also found to be significantly up or downregulated (Table 3), including two that followed the expression patterns of their antisense transcripts, namely *HOTAIRM1* (*HOXA* antisense transcript), and *FENDRR* (*FOXF1* adjacent non-coding developmental regulatory RNA). The transcriptional signatures induced by *Bb* suggest that the biological program of astrocytes is being changed by infection with the spirochete, and may reflect changes in the signaling environment or other complex biological parameters that undergo a change in response to the infection.

**Table 3:** Selected long non-coding RNAs altered in response to *Bb*.

		24h		48h	
Gene symbol	Gene Name	FC	p-value	FC	p-value
FENDRR	FOXF1 adjacent non-coding developmental regulatory RNA	4.37	1.06E-09	7.24	6.63E-18
HCP5	HLA complex P5 (non-protein coding)	-5.19	5.51E-29	-4.06	1.01E-22
HOTAIRM1	HOXA transcript antisense RNA, myeloid-specific 1	-9.21	5.37E-38	-11.67	2.10E-41
LINC-PINT	long intergenic non-protein coding RNA, p53 induced transcript	4.91	7.85E-27	4.73	1.59E-25
LINC00312	long intergenic non-protein coding RNA 312	-5.58	1.43E-06	-3.78	1.35E+04
LINC00842	long intergenic non-protein coding RNA 842	11.02	7.47E-25	22.41	1.27E-51
LINC01111	long intergenic non-protein coding RNA 1111	5.64	1.77E-24	5.28	4.48E-22
SNHG5	small nucleolar RNA host gene 5 (non-protein coding)	7.69	1.03E-169	6.87	1.10E-152
PLCE1-AS1	PLCE1 antisense RNA 1	2.21	9.99E+04	5.77	-0.009419462
APCDD1L-AS1	APCDD1L antisense RNA 1 (head to head)	-2.10	5.55E+00	-4.57	3.88E-14
LMCD1-AS1	LMCD1 antisense RNA 1 (head to head)	-3.57	6.60E-10	-5.17	6.07E-14
H19	H19, imprinted maternally expressed transcript (non-protein coding)	2.86	4.88E-06	4.18	8.98E-10

To confirm whether transcripts upregulated in response to *Bb* resulted in protein production and secretion after co-incubation with primary human astrocytes, we measured synthesis of selected proteins in astrocyte supernatants by ELISA (Fig 2B–2G). There were no differences in secreted factors at 48 hours post-*Bb* treatment (data not shown). However, as protein translation lags behind RNA production, we also measured selected proteins at 72 hours after *Bb* stimulation. Angiopoietin-like4 (ANGPTL4), Serpin Family G Member 1 (SERPING1), Chemokine (C-X-C Motif) Ligand 1 (CXCL-1), Chemokine (C-X-C Motif) Ligand 6 (CXCL-6), Dickkopf-related protein 1 (DKK1), and Stanniocalcin-1 (STC-1) were all induced at higher levels in *Bb*-treated astrocytes compared to untreated controls (Fig 2B–2G), which correlated with the observed changes in the respective transcripts for these genes by RNA-seq and RT-PCR.

### MicroRNA changes in astrocytes following *Bb* infection

**Fig 3:**
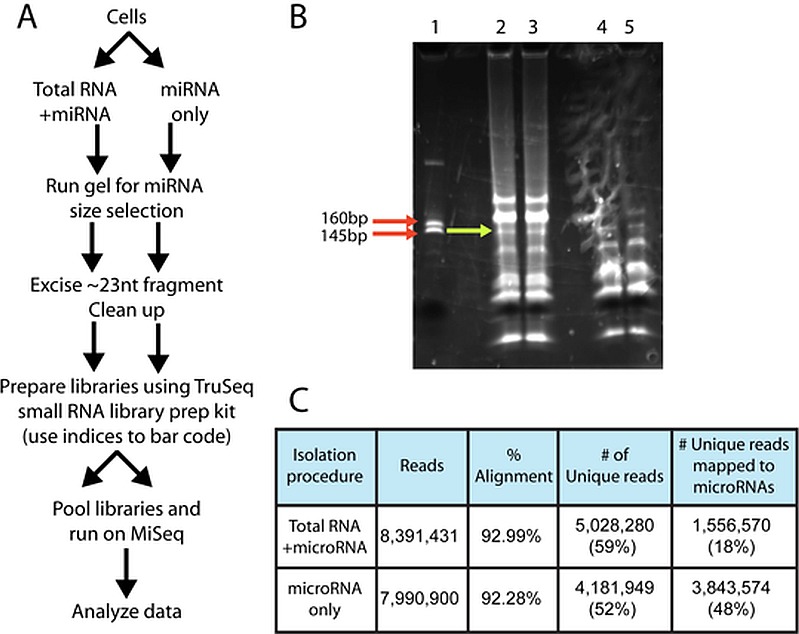
The microRNA-only isolation method for sequencing resulted in a higher percentage of unique reads that mapped to microRNAs. Astrocytes treated with *Bb* were lysed, and either total RNA and microRNA, or the microRNA-only fractions were isolated using procedures according to the miRNeasy kit (A). The microRNA from both preparations and libraries made using the Illumina TruSeq small RNA library kit. (B). Libraries were size-selected using the Illumina custom RNA ladder for size selection of the ~145-160bp band, and sequenced using the MiSeq. Lane 1: small RNA marker; lanes 2–3: duplicates of total RNA+miRNA preps; lanes 4–5: duplicates of miRNA only preps (C). The total number of reads, the percent alignments and number of unique reads were comparable in both conditions, but the microRNA-only fraction had a significantly higher percentage of unique reads that mapped to microRNAs relative to the total RNA+microRNA fraction (48% vs 18%). See text for details.

MicroRNAs are a class of small, non-coding RNAs between 21-24nt in length and have been shown to affect gene expression [39]. In order to investigate whether *Bb* infection causes changes in microRNA levels, we used small-RNA sequencing of microRNAs from astrocytes infected with *Bb* for 0, 24 and 48 hours. We isolated microRNAs using two methods based on the Qiagen miRNeasy kit (Fig 3A); either the microRNA fraction by itself, which enriches the population of microRNAs, or by purifying total RNA and microRNA, which is recommended by several groups for sequencing or microarray purposes. We then size-selected microRNA libraries (Fig 3B) for sequencing on the Illumina MiSeq. Although the recovery amounts were much greater for the total RNA+microRNA fraction relative to the microRNA only fraction (about a tenth of the total fraction), and the total number of sequencing reads as well as % alignments and unique reads were very comparable between the two fractions (Fig 3C), we determined that the microRNA only fraction actually returned more unique reads that mapped specifically to microRNAs (48% versus 18%) relative to the total RNA+miRNA fraction. Therefore, we used the microRNA only fraction to make libraries and perform the microRNA sequencing for the experiments described here.

**Fig 4:**
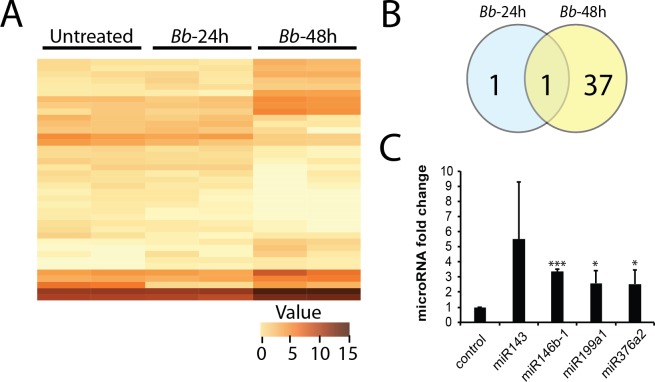
Differential expression of microRNAs following exposure to *Bb*. MicroRNAs were isolated from astrocytes treated with *Bb* for 24h (N = 3) and 48h (N = 3), using the microRNA-only procedure at the same time as RNAs, as described in Figs 1 and 3. Heat map analysis (A) showing changes in a subset of microRNAs (B) following 48h of *Bb* treatment. (C) Validation of microRNA changes were performed on 4 microRNAs (see text for details), and revealed upregulation of miR143, miR146b-1, miR199a1 and miR376a2. T-test (* = p-value<0.05; *** = p-value <0.0001).

**Fig 5:**
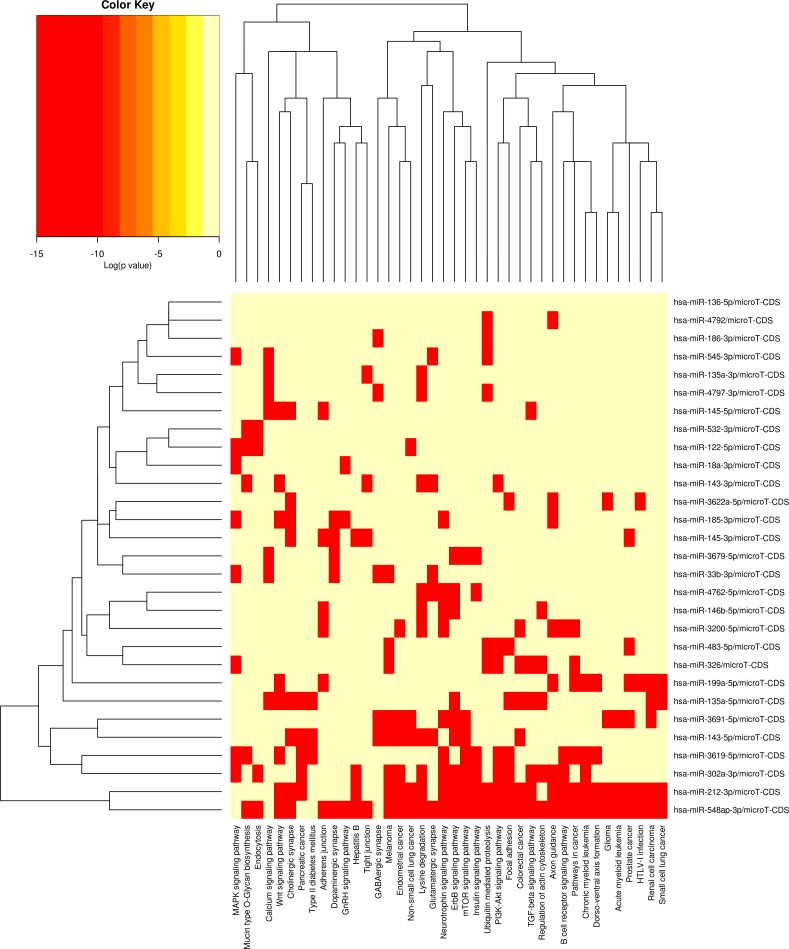
MicroRNA differential expression pathway analysis. We identified pathways targeted by differentially expressed miRNAs using the microT-CDS algorithm in DIANA-miRPath. Heat map of microRNAs vs Pathways, where microRNAs and pathways are clustered using Euclidean distances and complete linkage of binary values (0 = non-significant p-value and 1 = significant p-value). Red squares signify a significant p-value and light yellow signify a non-significant p-value.

Following isolation of microRNA only fractions from untreated (N = 3), *Bb* treated (N = 3; 24h) and *Bb* treated (N = 3; 48h) samples, we prepared pooled libraries using the TruSeq small RNA library preparation kit (Illumina) according to the manufacturer’s instructions, and sequenced the libraries on the MiSeq. A small subset of microRNAs were found to change significantly following 24h (2 microRNAs) and 48h (38 microRNAs) of infection (Fig 4A and 4B and S4 File). Using the DIANA microT-CDS algorithm [40], we found that several of the microRNAs that were differentially expressed were involved in signaling pathways, including PI3K-AKT, calcium signaling, and MAPK signaling pathways. MicroRNAs involved in cell adhesion, such as adherens junctions were also found to be affected (Fig 5). We validated some of these microRNAs using the miR-VILO kit from Life Technologies, and found that miR146b-1, miR199a1 and miR376a2 were significantly upregulated following 48h of *Bb* infection (Fig 4C). Another microRNA, miR143-3p was also upregulated, but failed to reach statistical significance as it appears to fluctuate significantly from treatment to treatment (Fig 4C). Of these microRNAs, hsa-miR-143-3p has been identified in plasma microRNA in chronic fatigue syndrome/myalgic encephalomyelitis [41], and hsa-miR-146b-5p was shown to have a role in stem cell differentiation processes [42].

To determine whether any known targets for these miRNAs are also altered, we used IPA analyses to compare changes in miRNA expression with simultaneous changes in our RNA-seq dataset (Table 4). Indeed, most microRNAs that were differentially expressed also corresponded with changes in expression of their predicted potential mRNA targets (15 in the case of miR-214-3p, for instance). Indeed, 58.5% of differentially expressed genes in this study were predicted to serve as targets for at least one of the differentially expressed microRNAs. The functional importance and relevance of these microRNAs remains to be seen, and the question of whether these are significantly differentially regulated in all cell types infected with *Bb* is still to be determined.

**Table 4:** Pairwise comparison of microRNA and RNA changes.

**microRNAs upregulated**	**# of DE targets**^**a**^
miR-1-3p (and other miRNAs w/seed GGAAUGU)	19
miR-132-3p (and other miRNAs w/seed AACAGUC)	11
miR-133a-3p (and other miRNAs w/seed UUGGUCC)	15
miR-135a-5p (and other miRNAs w/seed AUGGCUU)	14
miR-143-3p (and other miRNAs w/seed GAGAUGA)	13
miR-145-5p (and other miRNAs w/seed UCCAGUU)	11
miR-146a-5p (and other miRNAs w/seed GAGAACU)	17
miR-199a-5p (and other miRNAs w/seed CCAGUGU)	21
miR-216a-5p (miRNAs w/seed AAUCUCA)	9
miR-4797-3p (miRNAs w/seed CUCAGUA)	5
miR-545-3p (miRNAs w/seed CAGCAAA)	12
**microRNAs downregulated**	**# of DE targets**^**a**^
miR-122-5p (miRNAs w/seed GGAGUGU)	6
miR-302a-3p (and other miRNAs w/seed AAGUGCU)	17
miR-3200-5p (miRNAs w/seed AUCUGAG)	9
miR-326 (and other miRNAs w/seed CUCUGGG)	25
miR-33b-3p (and other miRNAs w/seed AGUGCCU)	15
miR-3619-3p (and other miRNAs w/seed CAGCAGG)	24
miR-3622a-5p (miRNAs w/seed AGGCACG)	7
miR-3679-5p (and other miRNAs w/seed GAGGAUA)	9
miR-3691-5p (miRNAs w/seed GUGGAUG)	9
miR-4762-5p (miRNAs w/seed CAAAUCU)	7
miR-4792 (miRNAs w/seed GGUGAGC)	12
miR-483-5p (miRNAs w/seed AGACGGG)	5
miR-532-3p (miRNAs w/seed CUCCCAC)	23
miR-548a-3p (and other miRNAs w/seed AAAACCA)	8
miR-942-5p (and other miRNAs w/seed CUUCUCU)	13

^a^Number of differentialy expressed (DE) genes identified by RNA-seq that are predicted to serve as targets of the corresponding DE microRNA as determined by IPA’s pairwise comparison tool.

## Discussion

Transcriptome studies can provide valuable insights into the pathophysiological mechanisms of disease. To date, few studies have examined global differential gene expression induced by *Bb* in any cell type. In this work, we demonstrate for the first time an extensive dataset of the transcriptional changes, including mRNAs, long non-coding RNAs, and microRNAs, induced by *Bb* in primary human astrocytes. 275 genes were differentially regulated in astrocytes co-cultured with *Bb*. Consistent with previous reports on *Bb*-induced gene expression, we observed alterations in expression of immune response genes including the chemokine genes *cxcl1*, *cxcl6*, and *cxcl8*, as well as *il1β* [16, 28, 31–33]. Other genes involved in inflammation and infection control that have not previously been linked to *Bb* infection were observed as well, most notably tumor necrosis factor superfamily member *tnfsf18* and *chi3l1*. TNFSF18 (GITRL) modulates T lymphocyte survival, and both TNFSF18 and its cognate receptor have been implicated in a number of inflammatory and autoimmune diseases in both human patients and experimental models of systemic lupus erythematosus, autoimmune encephalomyelitis, arthritis, and autoimmune diabetes [43–46].

Chitinase 3-like-1 (Chi3l1) is a secreted glycoprotein expressed by many cell types including stromal cells, activated macrophages, neutrophils, activated microglia, and reactive astrocytes [47–50]. Dysregulation of *chi3l1* has been reported for a number of human diseases characterized by acute or chronic inflammation and tissue remodeling (reviewed in [47]). Notably, *chi3l1* overexpression has been reported specifically in astrocytes associated with reactive gliosis in different acute and chronic neuropathological conditions; particularly those associated with neuroinflammation including multiple sclerosis, encephalitis, schizophrenia, Alzheimer's, ALS, and stroke models [50]. Additionally, it was shown that this overexpression is more abundantly associated with astrocytes in regions of inflammatory cells. Interestingly, expression of CHI3L1 has been suggested to enhance bacterial adhesion and invasion into tissues via binding to bacterial chitin-binding proteins (CBPs) in both inflammatory bowel disease and burn models [51–53]. This is intriguing, as *Bb* is an arthropod-transmitted bacterium that is suggested to encode CBPs [54], and has been well characterized to utilize host proteins to facilitate tissue binding and invasion [55, 56]. Notably, Chi3I1 promotes bacterial resistance and host tolerance during pneumococcal pneumonia, and mice deficient in *chi3I1* succumb more quickly to *Pseudomonas aeruginosa* infection [57, 58]. The significance of CHI3L1 during *Bb* infection is not known, and is currently the subject of further investigation.

We noted several genes involved in developmental processes were over-represented compared to other biological pathways. As astrocytes are key responders to insults in the CNS, play important roles in neurogenesis, and can be induced to form cancer-like stem cells, activation of developmental pathways suggests the intriguing possibility of reprogramming of astrocytes in response to infection [59, 60]. Of particular interest are the number of transcription factors, especially the homeobox and forkhead proteins, induced by *Bb*. Because transcription factors control expression of gene networks, changes in these factors might lead to significant downstream changes in cell states.

MicroRNAs have been implicated as important mediators of both Lyme arthritis [61] and Lyme carditis [62], however little is known about the importance of microRNAs for the pathophysiology of neuroborreliosis. We therefore profiled changes in the expression of microRNAs in response to *Bb* in astrocytes. To our knowledge, this is the first report of genome-wide changes in microRNA expression in response to *Bb* infection using a model of neuroborreliosis. Recent work identified upregulation of miR-146a, a key regulator of NF-κB signaling in the joints of *Bb*-infected mice [61]. This upregulation was limited to the joint and had no effect on bacterial clearance or inflammation in other tissues, while miR-155 played a role in Lyme carditis, but had little effect on joint inflammation [62]. While we did not find changes in the expression of hsa-mir-146a or hsa-mir-155 in astrocytes, we did find 38 microRNAs with altered expression. Altered microRNA expression has been implicated in a number of disease processes, including inflammatory CNS disease [63–67]. MicroRNAs known to be implicated in inflammation and cell function in another glial cell type, microglia, were observed, including miR145 (IL4/STAT6 signaling) and miR146b (NF-κB and JAK-STAT signaling) [68]. Notably, several differentially expressed microRNAs identified in this study have been identified in other models of CNS disorders/disease. Bai et al. previously demonstrated that increased expression of miR-143-3p led to decreased tight-junction protein expression and compromised blood-brain-barrier integrity in response to methamphetamine treatment, and that silencing of this microRNA using an antagomir was protective against these effects [69]. Elevated miR-143 has also been identified in plasma microRNA isolated from chronic fatigue syndrome/ encephalomyelitis patients [41]. Additionally, dysregulated miR-135 has been associated with altered anxiety and depression-like behavior, as well as altered responses to antidepressant treatments in mice [70]. As there is substantial overlap between these conditions and the constellation of symptoms often reported in patients with PTLDS or “chronic Lyme disease”, these microRNAs may have utility as biomarkers for complex conditions where there is a disturbance of both immune and nervous systems, and could serve as therapeutic targets where a causal link has been established [71, 72].

It is important to note that current evidence suggests that the damage caused by *Bb* is driven primarily by the host response to infection, not directly by toxins or other bacterial-produced factors. Understanding the host response is therefore key, and transcriptome studies are a small part of putting together the puzzle of *Bb* pathogenesis, host response, and potential long-term sequelae.

## Conclusions

We identified 275 RNAs and 38 microRNAs differentially expressed in human astrocytes in response to the Lyme disease spirochete, *Bb*. The identified genes include both previously characterized and novel gene expression changes associated with *Bb* infection. The expression changes of these RNAs and microRNAs could in part provide an explanation for the persistence of Lyme disease symptoms. Understanding how these changes are maintained over time will be of great importance in developing effective treatments to Lyme disease.

## Materials and Methods

### Bacteria

*B*. *burgdorferi* strain B31 MI-16 is an infectious clone of the sequenced type strain [73, 74] which contains all parental plasmids [75]. Bacteria were grown at 34°C to cell densities of approximately 1 × 10^7^ bacteria/ml in modified Barbour-Stoenner-Kelly (BSK-II) medium supplemented with 6% rabbit serum [76]. Plasmid content was monitored by multiplex PCR as described by Bunikis et al [77].

### Primary cultures of human astrocytes

Primary cultures of human astrocytes were obtained from ScienCell Research Laboratories (Carlsbad, CA; catalog #1800) and maintained on poly-L-lysine coated flasks (2 mg/cm^2^, T-75) in astrocyte medium containing antibiotics penicillin (1,000 units/mL) and streptomycin (1,000 μg/mL) (ScienCell, catalog # 1801). To stimulate the cells, astrocytes were used at passage 3 at approximately 85–90% confluence. Prior to *Bb* stimulation, cells were washed 3x with sterile Dulbecco’s phosphate buffered saline (DPBS) and the medium was replaced with antibiotic-free astrocyte medium. Mean astrocyte density in one representative T75 was determined using an automated cell counter (Invitrogen). Astrocytes were then stimulated with *Bb* at a multiplicity of infection (MOI) of 10:1 for 24 or 48 hours. Non-treatment control flasks were prepared in an identical fashion with the absence of *Bb* infection. Astrocyte viability and adhesion were monitored by light microscopy; at the multiplicity of infection used, *Bb* had no impact on astrocyte viability (data not shown).

### MicroRNA and RNA isolation and cDNA synthesis

MicroRNAs and RNA were simultaneously isolated from the same dish of cells using the miRNeasy kit (Qiagen, Valencia, CA) according to the manufacturer’s instructions. Briefly, after aspiration of media, cells were directly lysed by addition of QIAzol lysis reagent, detached using cell lifters, homogenized and transferred to an RNase-DNase free Eppendorf tube. Cells were treated with chloroform for 2–3 minutes and centrifuged at 12,000 x g for 15 minutes at 4°C. The upper aqueous phase was mixed with 70% ethanol and spun through a Qiagen column. The flow-through was retained and processed for microRNAs, and the column (which contained the RNA fraction) was processed separately, thus yielding both RNA and microRNA from the same dish. Genomic DNA was removed by DNA digestion with RNase-Free DNase Set (catalog # 79254, Qiagen). RNA quality and concentration was assessed using a spectrophotometer (NanoDrop), and by electrophoresis on a 2% agarose gel. MicroRNA quality was assessed using the NanoDrop and by electrophoresis on a 15% NuPAGE gel.

### Library construction, microRNA- and RNA-sequencing

The total RNA isolated as described above was used for 50 bp single-end RNA-Sequencing at the University of Minnesota Genomics Center (UMGC) on the Illumina HiSeq 2000. The quality was assessed by the Agilent Bioanalyzer at their facility and samples with high RNA integrity number (>8) were used for library construction following the manufacturer’s (Illumina) instructions. In summary, 1 microgram of total RNA was oligo-dT purified using oligo-dT coated magnetic beads, chemically fragmented and then reverse transcribed into cDNA. The cDNA was fragmented, blunt-ended, and ligated to indexed (barcoded) adaptors and amplified using 15 cycles of PCR. Final library size distribution was validated using capillary electrophoresis and quantified using fluorimetry (PicoGreen) and via Q-PCR. Indexed libraries were normalized, pooled and then size selected to 320bp +/- 5% using Caliper’s XT instrument. TruSeq libraries were hybridized to a single end flow cell and individual fragments clonally amplified by bridge amplification on the Illumina cBot. Once clustering was complete, the flow cell was loaded on the HiSeq 2000 and sequenced using Illumina’s SBS chemistry. Three biological replicates for each time point of *Bb* treatment were sequenced, resulting in an average of 50 million reads per sample. Base call (.bcl) files for each cycle of sequencing were generated by Illumina Real Time Analysis (RTA) software. The base call files and run folders were then exported to servers maintained at the Minnesota Supercomputing Institute. Primary analysis and de-multiplexing were performed using Illumina’s CASAVA software 1.8.2. The end result of the CASAVA workflow is de-multiplexed FASTQ files that were subject to subsequent analyses as described below.

For micro-RNA sequencing, libraries were prepared in-house and run on the MiSeq at the UND Epigenomics core. Briefly, the TruSeq small RNA sample prep kit (Illumina) was used to add the 3’ and 5’ adapters, reverse transcribe, and amplify the miRNAs from two biological replicates per time point using barcodes as before for each sample. The libraries were purified by excising the bands corresponding to the microRNA fraction (roughly between 145 and 160bp, using the custom RNA ladder from Illumina) on a 15% NuPAGE gel. Libraries were then validated, pooled, and sequenced on the Illumina MiSeq using 50 bp single-end reads, resulting in approximately 2 million reads per sample.

### Data analysis

#### RNA data analysis

Preliminary quality control analysis of the FASTQ files was performed using FastQC v0.11.2 [78]. Reads were aligned to the human genome (hg19) using Tophat v2.0.13 [79]. Fragments were assigned to genes using HTSeq v0.6.1p1 [80]. Differential expression analysis was performed using EdgeR [81], with the FDR controlled at 0.05. Clustering of significant genes was performed using log2(cpm) values. Network mapping and functional analyses were generated through the use of both PANTHER and QIAGEN’s Ingenuity Pathway Analysis (IPA®, QIAGEN Redwood City, www.qiagen.com/ingenuity). RNA fastq files have been submitted to the NCBI Gene Expression Omnibus (GEO) database [82] with experiment series accession number [GSE85143].

#### MicroRNA data analysis

Preliminary quality control analysis of the 9 FASTQ files was carried out using FastQC v0.11.2 [78]. Reads were trimmed by removing the 5’ Small RNA Sequencing Primer and the 3’ RNA adapter using Cutadapt v.1.6 (Martin, 2011). Reads which were shorter than 18nt after trimming were removed. Trimmed reads were further filtered by removing low quality reads with a Phred score <20. Finally, reads which were longer than 27nt were removed. Remaining reads were processed using the mirDeep v2.0.0.5 [83] software package. The mapper module was used to map reads to the human genome (hg19) using Bowtie v.1.1.1 [84]. The quantifier module was used to map the reads to known miRBase v21[85] precursors and determine expression of corresponding miRNAs. Novel and known miRNAs were identified using the miRDeep2 module. The R/Bioconductor package edgeR v3.8.6 [81] was used to identify differentially expressed miRNAs. MicroRNA pathway analysis was performed using DIANA-miRPath v.2.0 [86] using the microT-CDS database for predicted miRNA targets. MicroRNAs vs Pathways heat maps were generated using the “Pathways Union” and “Targeted Pathways Clusters” options. Venn diagrams of differentially expressed miRNAs, and associated pathways and genes were constructed using the R package VennDiagram [87]. miRNA fastq files have been submitted to the NCBI Gene Expression Omnibus (GEO) database [82] with experiment series accession number [GSE85142]. Data were analyzed through the use of QIAGEN’s Ingenuity® Pathway Analysis (IPA®, QIAGEN Redwood City, www.qiagen.com/ingenuity). The networks, functional analyses, and comparison to the RNA targets of miRNAs were generated through the use of QIAGEN’s Ingenuity Pathway Analysis (IPA®, QIAGEN Redwood City, www.qiagen.com/ingenuity).

The datasets generated during and/or analyzed during the current study are available in the GEO repository, with accession numbers GSE85143 (RNA-seq), and GSE85142 (miRNA-seq). Any additional information and/or materials will be available from the corresponding authors on reasonable request.

### Validation of selected transcripts

Changes in individual genes were confirmed using individual PCR primer sets (Qiagen Quantitect primers). Briefly, each reaction contained 6 μl nuclease-free H_2_O, 2 μl primer mix at 10 μM, 10 μl BioRad SYBRGreen Supermix and +/- 2 μl template DNA or water (no template control). The qPCR was performed in 40 cycles following an initial 2 min denaturation at 95°C. Each cycle consisted of a 1 min annealing step performed at 60°C, followed by a 15-sec melting interval at 95°C. Product melting curves were generated at the end of the reaction using a stepped temperature gradient of 0.5°C x 10 sec starting at 60°C. Expression levels of all transcripts were compared to housekeeping gene (GAPDH) and the relative changes in gene expression were compared to those of untreated cells using the 2^-ΔΔCT^ method where C_T_ = threshold cycle. This method was used on each individual example with the untreated sample as the comparator (e.g., ΔΔC_T_ = ΔC_T_ (experimental)–ΔC_T_ (control)) [29]. All samples were analyzed in triplicate from three independent biological replicates per time point. *Bb*-infected samples were compared to uninfected controls as determined by one-way ANOVA followed by Holm-Sidak comparison to control group. Groups were considered significantly different from control samples if p<0.05.

### Enzyme-linked immunosorbent assays

Culture supernatants were removed after stimulation and stored at -80°C. ELISA for ANGPTL4, CXCL1, CXCL6, DKK1, SERPING1, STC1, TGFA, and THBD was performed according to the manufacturer’s instructions (R&D Systems, Minneapolis, MN). Briefly, all reagents were brought to room temperature and prepared as instructed. Plates were coated overnight with 100 μl of appropriate capture antibody. Following aspiration and wash, 100 μl of appropriate chemokine standards, controls, or sample were added to each well. Plates were incubated for 2 hours at room temperature. Following aspiration and wash, 100 μl of antibody conjugate was added to each well, followed by a 2-hour incubation at room temperature. Following aspiration and washes, the chemokine of interest was detected by adding a chromogenic substrate followed by a stop solution. Plates were read at an optical density of 450 nm on a BioTek Epoch plate reader. Samples were run in triplicate and data pooled from each treatment group. *Bb*-infected samples were compared to uninfected controls as determined by Student’s t-test. Groups were considered significantly different from control samples if p<0.05.

## Supporting Information

S1 Fig Multidimensional scaling (MDS) plot of RNA-seq data. MDS plots were created using edgeR from the RNA-seq data from the untreated and *Bb* treatments for each day. The plots indicated that the replicates clustered together by treatment groups with no outliers. Untreated samples are seen in green, 24h treatments in black and 48h treatments in red.
(TIFF)

S2 Fig IPA canonical pathways. Genes that are significantly altered following *Bb* treatment were uploaded to the Ingenuity Pathway Analysis website and were analyzed by their proprietary software, which classified the genes into distinct pathways.
(PNG)

S1 File Significant differential expression gene list (RNAs). Transcripts that were significantly different (FDR of 0.05 or below, four-fold change) between the untreated and the *Bb* treated groups are listed.
(XLSX)

S2 File IPA core analysis. Ingenuity Pathway Analysis generated a core analysis using RNA sequencing data that we uploaded to their website.
(PDF)

S3 File IPA networks. The RNA-seq expression value changes (S1 File) were uploaded to the Ingenuity Pathway Analysis website and were analyzed by their proprietary software. The IPA Network Generation Algorithm created these networks. Top functions of the genes were related to cellular assembly and organization, connective tissue development and function, neurological disease. Node (gene) and edge (gene relationship) symbols are described in the key on the last page after the network images. The intensity of the node color indicates the degree of upregulation (red), downregulation (green) or uncolored (grey). Genes in uncolored nodes were not identified as differentially expressed in our experiment, and were integrated into the computationally generated networks on the basis of the evidence stored in the IPA knowledge memory indicating a relevance to this network. The node shapes denote enzymes, phosphatases, kinases, peptidases, G-protein coupled receptor, transmembrane receptor, cytokines, growth factor, ion channel, transporter, translation factor, nuclear receptor, transcription factor and other (key).
(PDF)

S4 File Significant differential expression gene list (microRNAs). List of microRNAs that were significantly different (p-value of 0.01 or below, four-fold change) between the untreated and the *Bb* treated groups.
(XLSX)
