# Supplementary figures and images for: MicroRNA and mRNA Transcriptome Profiling in Primary Human Astrocytes Infected with Borrelia burgdorferi
Source: PLoS One. 2017 Jan 30;12(1):e0170961. doi: 10.1371/journal.pone.0170961 (PMC5279786; doi:10.1371/journal.pone.0170961)

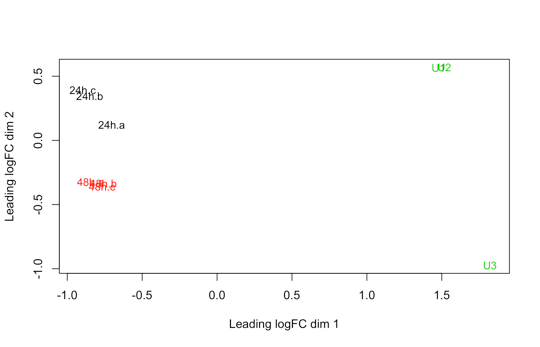

Supplement: S1 Fig — MDS plots were created using edgeR from the RNA-seq data from the untreated and Bb treatments for each day. The plots indicated that the replicates clustered together by treatment groups with no outliers. Untreated samples are seen in green, 24h treatments in black and 48h treatments in red. (TIFF) [file pone.0170961.s001.tiff]

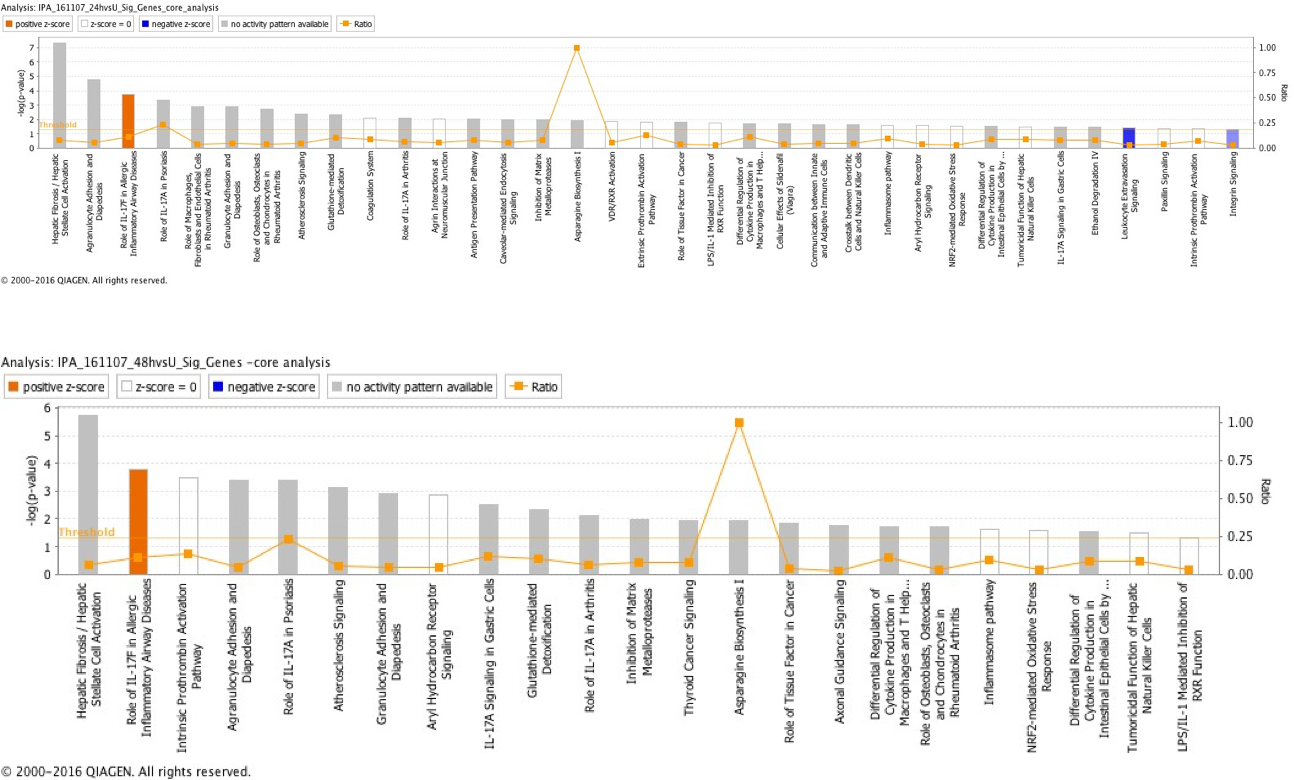

Supplement: S2 Fig — Genes that are significantly altered following Bb treatment were uploaded to the Ingenuity Pathway Analysis website and were analyzed by their proprietary software, which classified the genes into distinct pathways. (PNG) [file pone.0170961.s002.png]
