# Supplementary material for: MicroRNA and mRNA Transcriptome Profiling in Primary Human Astrocytes Infected with Borrelia burgdorferi
Source: PLoS One. 2017 Jan 30;12(1):e0170961. doi: 10.1371/journal.pone.0170961 (PMC5279786; doi:10.1371/journal.pone.0170961)
Supplement: S2 File — Ingenuity Pathway Analysis generated a core analysis using RNA sequencing data that we uploaded to their website. (PDF) [file pone.0170961.s004.pdf]

INGENUITY
PATHWAY ANALYSIS

Analysis Name: IPA_161107_24hvsU_Sig_Genes_core_analysis
Analysis Creation Date: 2016-11-17
Build version: 400896M
Content version: 28820210 (Release Date: 2016-09-24)

Analysis Settings

Reference set: Ingenuity Knowledge Base (Genes Only)

Relationship to include: Direct and Indirect

Includes Endogenous Chemicals

Optional Analyses: My Pathways My List

Filter Summary:

Consider only relationships where

confidence = Experimentally Observed

(c) 2000-2016 QIAGEN. All rights reserved.

Top Canonical Pathways

Name	p-value	Overlap
Hepatic Fibrosis / Hepatic Stellate Cell Activation	4.51E-08	7.7 % 14/183
Agranulocyte Adhesion and Diapedesis	1.78E-05	5.8 % 11/189
Role of IL-17F in Allergic Inflammatory Airway Diseases	1.73E-04	11.4 % 5/44
Role of IL-17A in Psoriasis	4.40E-04	23.1 % 3/13
Role of Macrophages, Fibroblasts and Endothelial Cells in Rheumatoid Arthritis	1.28E-03	3.6 % 11/309

Top Upstream Regulators

Upstream Regulator	p-value of overlap	Predicted Activation
TNF	3.41E-14	
HGF	2.18E-13	
IFNG	9.77E-13	
dexamethasone	1.14E-12	
Vegf	9.30E-12	Activated

Top Diseases and Bio Functions

Diseases and Disorders

Name	p-value	#Molecules
Cancer	2.05E-03 - 1.37E-12	244
Organismal Injury and Abnormalities	2.19E-03 - 1.37E-12	248
Dermatological Diseases and Conditions	1.91E-03 - 5.23E-12	149
Gastrointestinal Disease	2.05E-03 - 2.24E-10	221
Reproductive System Disease	1.67E-03 - 2.76E-08	148

Molecular and Cellular Functions

(c) 2000-2016 QIAGEN. All rights reserved.

Name	p-value	#Molecules
Cellular Movement	2.19E-03 - 4.90E-11	80
Cellular Growth and Proliferation	2.06E-03 - 5.99E-10	124
Cellular Development	2.06E-03 - 1.58E-07	100
Cell-To-Cell Signaling and Interaction	2.06E-03 - 5.20E-07	63
Lipid Metabolism	2.01E-03 - 4.35E-06	37

Physiological System Development and Function

Name	p-value	#Molecules
Cardiovascular System Development and Function	2.19E-03 - 1.07E-13	69
Organismal Development	2.06E-03 - 2.71E-10	93
Embryonic Development	2.06E-03 - 8.82E-09	76
Skeletal and Muscular System Development and Function	2.06E-03 - 2.76E-07	62
Connective Tissue Development and Function	2.06E-03 - 4.31E-07	46

Top Tox Functions

Assays: Clinical Chemistry and Hematology

Name	p-value	#Molecules
Increased Levels of Albumin	6.95E-02 - 1.19E-02	2
Decreased Levels of Albumin	4.69E-02 - 4.69E-02	1
Increased Levels of ALT	8.06E-02 - 8.06E-02	1
Increased Levels of LDH	1.65E-01 - 1.65E-01	1
Increased Levels of Alkaline Phosphatase	2.12E-01 - 2.12E-01	2

Cardiotoxicity

(c) 2000-2016 QIAGEN. All rights reserved.

INGENUITY PATHWAY ANALYSIS

Name	p-value	#Molecules
Cardiac Dysfunction	2.83E-01 - 1.64E-03	8
Cardiac Infarction	3.62E-01 - 2.00E-03	10
Cardiac Fibrosis	2.14E-01 - 2.19E-03	5
Cardiac Arrythmia	3.74E-01 - 3.50E-03	8
Cardiac Stenosis	5.83E-02 - 8.65E-03	4

Hepatotoxicity

Name	p-value	#Molecules
Liver Hyperplasia/Hyperproliferation	3.35E-01 - 5.06E-04	103
Liver Inflammation/Hepatitis	2.04E-01 - 6.18E-04	9
Glutathione Depletion In Liver	1.65E-03 - 1.65E-03	3
Liver Cirrhosis	1.38E-01 - 1.19E-02	5
Liver Fibrosis	2.41E-01 - 1.96E-02	5

Nephrotoxicity

Name	p-value	#Molecules
Renal Proliferation	1.03E-01 - 1.85E-03	9
Renal Hypoplasia	3.54E-02 - 3.93E-03	4
Glomerular Injury	4.58E-01 - 5.99E-03	10
Renal Damage	3.03E-01 - 5.99E-03	7
Renal Necrosis/Cell Death	2.41E-01 - 8.27E-03	12

Top Regulator Effect Networks

ID	Regulators	Diseases & Functions	Consistency Score
1	KAT6A,RELA,SPP1	influx of neutrophils,transformation of vertebrae	3.889
2	CCL11,SPP1	binding of cells	3.13

(c) 2000-2016 QIAGEN. All rights reserved.

INGENUITY PATHWAY ANALYSIS

3	NFE2L2	binding of tumor cell lines	-7.506
4	Vegf	binding of tumor cell lines	-22.0

Top Networks

ID	Associated Network Functions	Score
1	Vitamin and Mineral Metabolism, Connective Tissue Development and Function, Lipid Metabolism	40
2	Tissue Development, Cardiovascular Disease, Organismal Injury and Abnormalities	38
3	Connective Tissue Development and Function, Connective Tissue Disorders, Nervous System Development and Function	31
4	Connective Tissue Disorders, Organismal Injury and Abnormalities, Cancer	27
5	Drug Metabolism, Glutathione Depletion In Liver, Protein Synthesis	25

Top Tox Lists

Name	p-value	Overlap
Hepatic Fibrosis	3.42E-06	8.9 % 9/101
Glutathione Depletion - Phase II Reactions	1.65E-03	15.0 % 3/20
Increases Heart Failure	2.49E-03	13.0 % 3/23
Increases Cardiac Dysfunction	3.42E-03	7.7 % 4/52
Acute Renal Failure Panel (Rat)	6.43E-03	6.5 % 4/62

Top Analysis-Ready Molecules

Exp Log Ratio up-regulated

Molecules	Exp. Value	Exp. Chart
**TNFSF18**	↑ 8.786	
**SIX6**	↑ 6.299	
**C14orf39**	↑ 6.061	
**SHOX2**	↑ 5.928	
**RNF5P1**	↑ 5.602	

(c) 2000-2016 QIAGEN. All rights reserved.

**GSTM1**	↑ 5.479	
**IL1B**	↑ 5.421	
**LYVE1**	↑ 5.297	
**LIPH**	↑ 5.204	
**CHI3L1**	↑ 4.712	

Exp Log Ratio down-regulated

Molecules	Exp. Value	Exp. Chart
**GSTT1**	↓ -11.037	
**CTAG2**	↓ -7.067	
**IGFL3**	↓ -6.550	
FKBP1AP1	↓ -6.393	
**HAPLN1**	↓ -6.271	
**PLN**	↓ -6.239	
**MMP13**	↓ -5.832	
**HOXD4**	↓ -4.858	
**RARRES2**	↓ -4.517	
**HOXA2**	↓ -4.459	

(c) 2000-2016 QIAGEN. All rights reserved.

INGENUITY PATHWAY ANALYSIS

Analysis Name: IPA_161107_48hvsU_Sig_Genes -core analysis
Analysis Creation Date: 2016-11-17
Build version: 400896M
Content version: 28820210 (Release Date: 2016-09-24)

Analysis Settings

Reference set: Ingenuity Knowledge Base (Genes Only)

Relationship to include: Direct and Indirect

Includes Endogenous Chemicals

Optional Analyses: My Pathways My List

Filter Summary:

Consider only relationships where

confidence = Experimentally Observed

(c) 2000-2016 QIAGEN. All rights reserved.

Top Canonical Pathways

Name	p-value	Overlap	
Hepatic Fibrosis / Hepatic Stellate Cell Activation	1.83E-06	6.6 %	12/183
Role of IL-17F in Allergic Inflammatory Airway Diseases	1.60E-04	11.4 %	5/44
Intrinsic Prothrombin Activation Pathway	3.50E-04	13.8 %	4/29
Agranulocyte Adhesion and Diapedesis	4.12E-04	4.8 %	9/189
Role of IL-17A in Psoriasis	4.20E-04	23.1 %	3/13

Top Upstream Regulators

Upstream Regulator	p-value of overlap	Predicted Activation
TNF	7.62E-14	
dexamethasone	1.92E-13	
tretinoin	8.53E-11	
IFNG	1.29E-10	
okadaic acid	2.15E-10	

Top Diseases and Bio Functions

Diseases and Disorders

Name	p-value	#Molecules
Cancer	9.60E-04 - 1.90E-10	238
Organismal Injury and Abnormalities	9.64E-04 - 1.90E-10	242
Dermatological Diseases and Conditions	8.63E-04 - 2.18E-10	142
Gastrointestinal Disease	9.60E-04 - 3.51E-09	214
Connective Tissue Disorders	9.64E-04 - 6.06E-09	65

Molecular and Cellular Functions

(c) 2000-2016 QIAGEN. All rights reserved.

INGENUITY PATHWAY ANALYSIS

Name	p-value	#Molecules
Cellular Growth and Proliferation	8.98E-04 - 3.33E-13	129
Cellular Movement	9.31E-04 - 4.40E-10	81
Cellular Development	9.64E-04 - 2.58E-09	115
Lipid Metabolism	5.05E-04 - 1.78E-07	44
Small Molecule Biochemistry	7.62E-04 - 1.78E-07	52

Physiological System Development and Function

Name	p-value	#Molecules
Cardiovascular System Development and Function	9.62E-04 - 2.73E-12	63
Embryonic Development	8.98E-04 - 5.17E-11	85
Organismal Development	9.62E-04 - 5.17E-11	119
Connective Tissue Development and Function	9.64E-04 - 6.06E-09	66
Nervous System Development and Function	9.31E-04 - 6.06E-09	70

Top Tox Functions

Assays: Clinical Chemistry and Hematology

Name	p-value	#Molecules
Increased Levels of Albumin	6.84E-02 - 1.17E-02	2
Decreased Levels of Albumin	4.62E-02 - 4.62E-02	1
Increased Levels of ALT	2.20E-01 - 7.94E-02	2
Increased Levels of LDH	1.62E-01 - 1.62E-01	1
Increased Levels of Red Blood Cells	3.33E-01 - 3.33E-01	2

Cardiotoxicity

(c) 2000-2016 QIAGEN. All rights reserved.

INGENUITY PATHWAY ANALYSIS

Name	p-value	#Molecules
Pulmonary Hypertension	3.48E-02 - 3.60E-04	7
Cardiac Dysfunction	2.77E-01 - 1.47E-03	8
Cardiac Fibrosis	2.11E-01 - 2.09E-03	6
Cardiac Congestive Cardiac Failure	2.29E-03 - 2.29E-03	6
Heart Failure	3.47E-01 - 2.29E-03	9

Hepatotoxicity

Name	p-value	#Molecules
Liver Inflammation/Hepatitis	5.42E-01 - 3.90E-05	10
Glutathione Depletion In Liver	1.57E-03 - 1.57E-03	3
Liver Hematopoiesis	5.81E-03 - 5.81E-03	2
Liver Hyperplasia/Hyperproliferation	3.31E-01 - 7.88E-03	95
Liver Cirrhosis	5.29E-02 - 1.17E-02	8

Nephrotoxicity

Name	p-value	#Molecules
Renal Dysplasia	3.48E-02 - 1.57E-03	3
Renal Proliferation	6.84E-02 - 1.74E-03	11
Renal Hypoplasia	3.48E-02 - 3.71E-03	4
Renal Damage	2.99E-01 - 4.82E-03	7
Renal Tubule Injury	4.82E-03 - 4.82E-03	5

Top Regulator Effect Networks

ID	Regulators	Diseases & Functions	Consistency Score
1	Akt,APOE,CYR61,Insulin,SPP1,WISP2	mammary tumor	1.94
2	RELA	mammary tumor	-4.95

(c) 2000-2016 QIAGEN. All rights reserved.

3	IL10	mammary tumor	-5.715

Top Networks

ID	Associated Network Functions	Score
1	Cancer, Connective Tissue Disorders, Organismal Injury and Abnormalities	36
2	Neurological Disease, Skeletal and Muscular Disorders, Cancer	32
3	Cell-To-Cell Signaling and Interaction, Cellular Assembly and Organization, Cellular Movement	24
4	Drug Metabolism, Glutathione Depletion In Liver, Protein Synthesis	23
5	Connective Tissue Development and Function, Connective Tissue Disorders, Organ Morphology	23

Top Tox Lists

Name	p-value	Overlap	
Hepatic Fibrosis	2.55E-05	7.9 %	8/101
Glutathione Depletion - Phase II Reactions	1.57E-03	15.0 %	3/20
Cytochrome P450 Panel - Substrate is a Vitamin (Human)	2.00E-03	33.3 %	2/6
Increases Heart Failure	2.38E-03	13.0 %	3/23
Cytochrome P450 Panel - Substrate is a Vitamin (Mouse)	2.78E-03	28.6 %	2/7

Top Analysis-Ready Molecules

Exp Log Ratio up-regulated

Molecules	Exp. Value	Exp. Chart
**TNFSF18**	↑ 9.187	
**SIX6**	↑ 6.823	
**C14orf39**	↑ 6.432	
ECEL1P2	↑ 6.209	
**LIPH**	↑ 6.016	
**RNF5P1**	↑ 5.715	
**SHOX2**	↑ 5.699	

(c) 2000-2016 QIAGEN. All rights reserved.

**GSTM1**	↑ 5.361	
**IL1B**	↑ 5.194	
**LYVE1**	↑ 5.098	

Exp Log Ratio down-regulated

Molecules	Exp. Value	Exp. Chart
**GSTT1**	↓ -10.944	
**CTAG2**	↓ -8.868	
FKBP1AP1	↓ -7.324	
**MMP13**	↓ -6.478	
**IGFL3**	↓ -6.015	
**HOXD4**	↓ -5.504	
**HAPLN1**	↓ -5.147	
**EN2**	↓ -4.793	
**HSD17B2**	↓ -4.218	
**HOXA3**	↓ -3.899	

(c) 2000-2016 QIAGEN. All rights reserved.

INGENUITY PATHWAY ANALYSIS
