# Supplementary material for: MicroRNA and mRNA Transcriptome Profiling in Primary Human Astrocytes Infected with Borrelia burgdorferi
Source: PLoS One. 2017 Jan 30;12(1):e0170961. doi: 10.1371/journal.pone.0170961 (PMC5279786; doi:10.1371/journal.pone.0170961)
Supplement: S3 File — The RNA-seq expression value changes (S1 File) were uploaded to the Ingenuity Pathway Analysis website and were analyzed by their proprietary software. The IPA Network Generation Algorithm created these networks. Top functions of the genes were related to cellular assembly and organization, connective tissue development and function, neurological disease. Node (gene) and edge (gene relationship) symbols are described in the key on the last page after the network images. The intensity of the node color indicates the degree of upregulation (red), downregulation (green) or uncolored (grey). Genes in uncolored nodes were not identified as differentially expressed in our experiment, and were integrated into the computationally generated networks on the basis of the evidence stored in the IPA knowledge memory indicating a relevance to this network. The node shapes denote enzymes, phosphatases, kinases, peptidases, G-protein coupled receptor, transmembrane receptor, cytokines, growth factor, ion channel, transporter, translation factor, nuclear receptor, transcription factor and other (key). (PDF) [file pone.0170961.s005.pdf]

Supplementary information File S3

Ingenuity Pathway Analysis (IPA) generated networks using transcripts that are differentially expressed between untreated and Bb-treated samples.

S3.1. Network of genes significantly differentially expressed in untreated vs. 24h samples.

Network 1. Vitamin and Mineral Metabolism, Connective Tissue Development and Function, Lipid Metabolism.

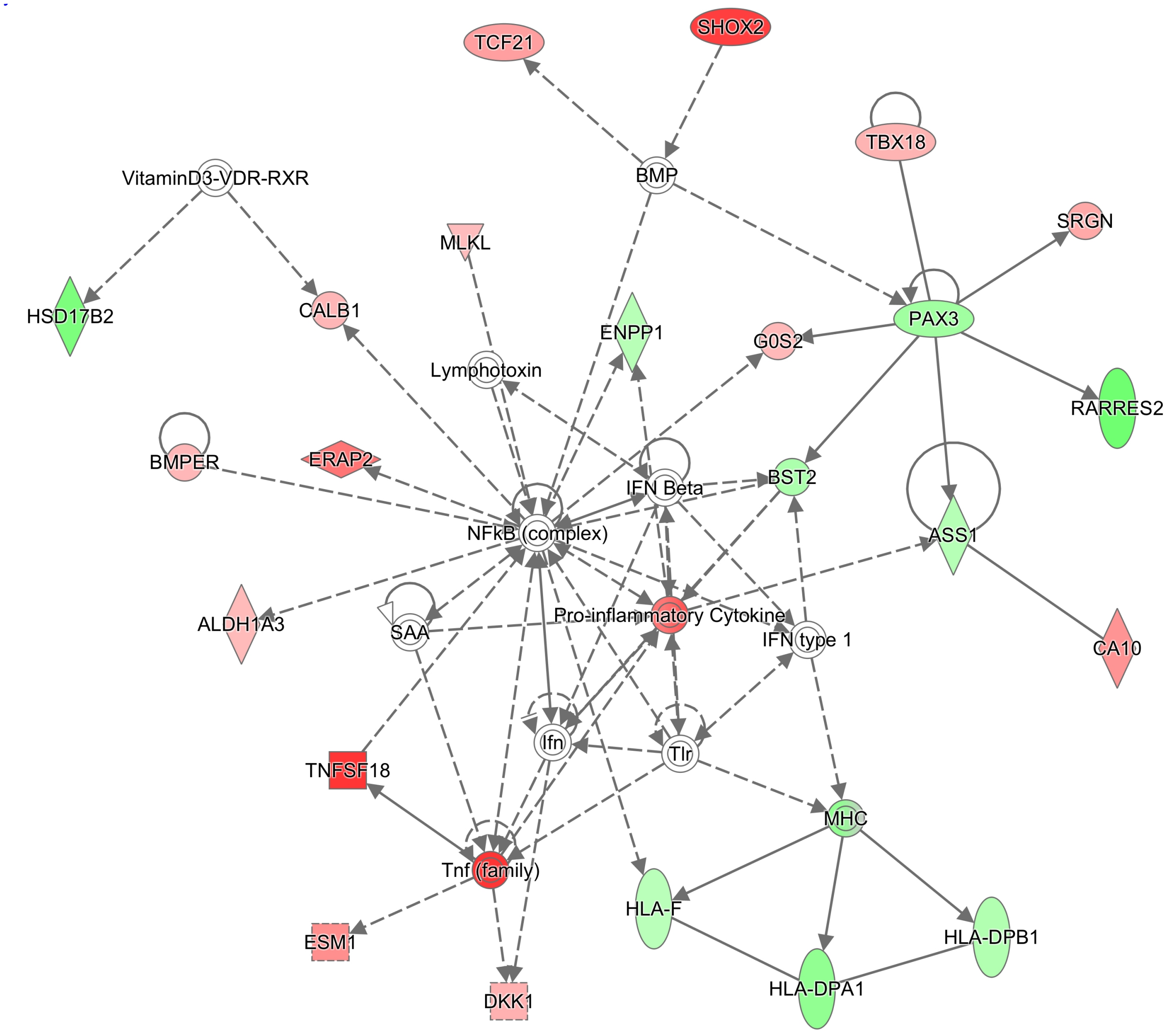

© 2000-2016 QIAGEN. All rights reserved.

Network 2. Tissue Development, Cardiovascular Disease, Organismal Injury and Abnormalities

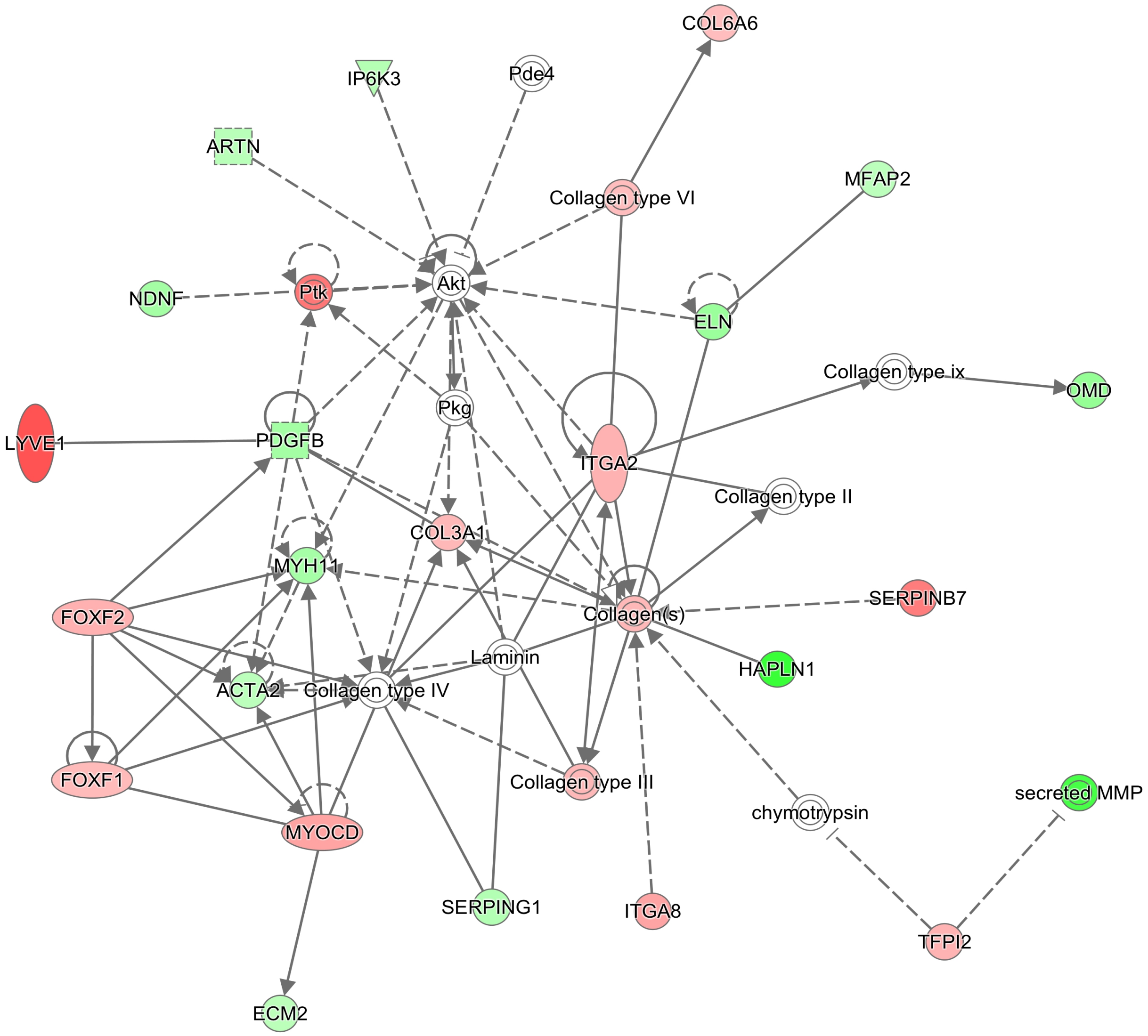

© 2000-2016 QIAGEN. All rights reserved.

Network 3. Connective Tissue Development and Function, Connective Tissue Disorders, Nervous System Development and Function

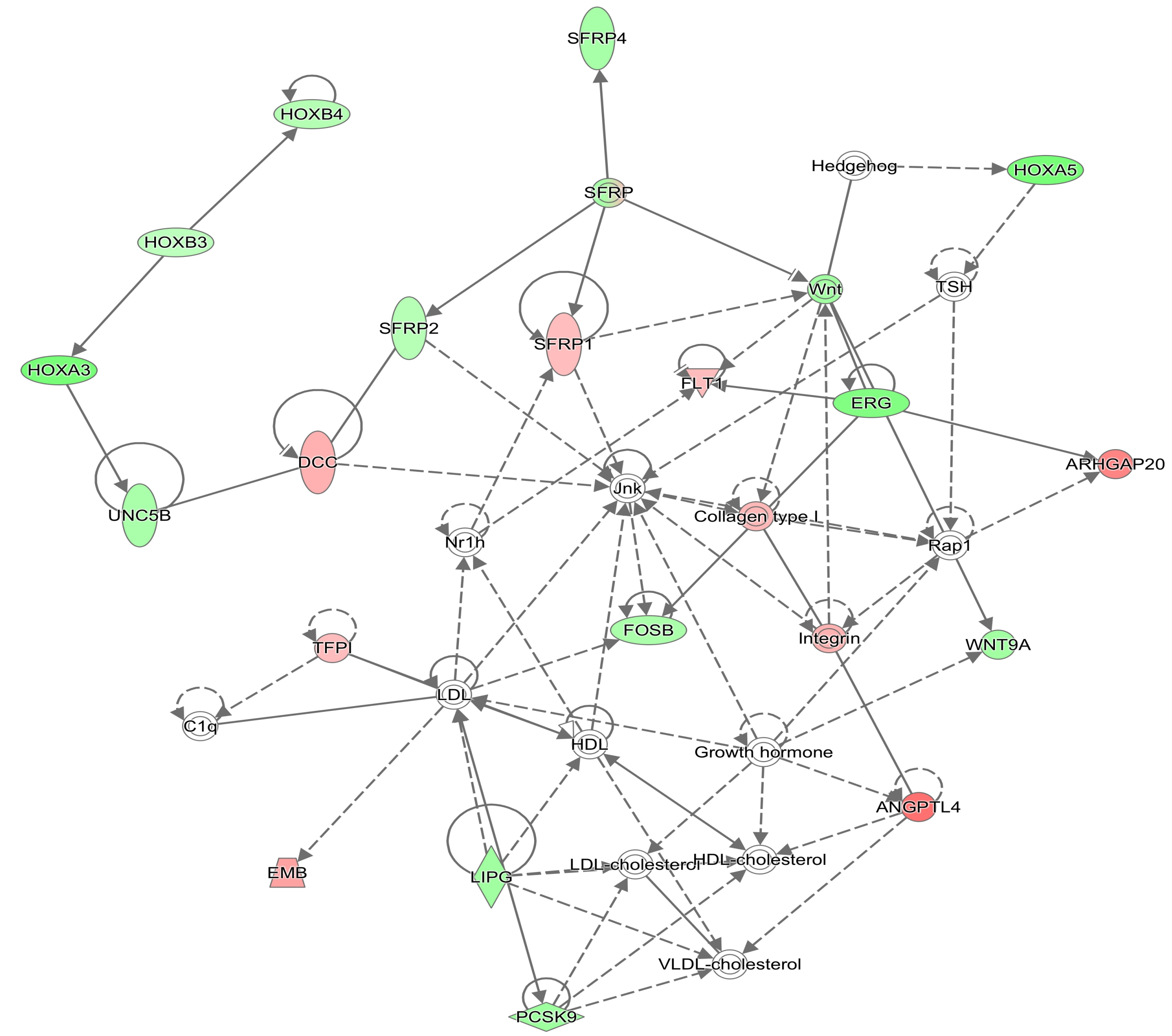

© 2000-2016 QIAGEN. All rights reserved.

Network 4. Connective Tissue Disorders, Organismal Injury and Abnormalities, Cancer

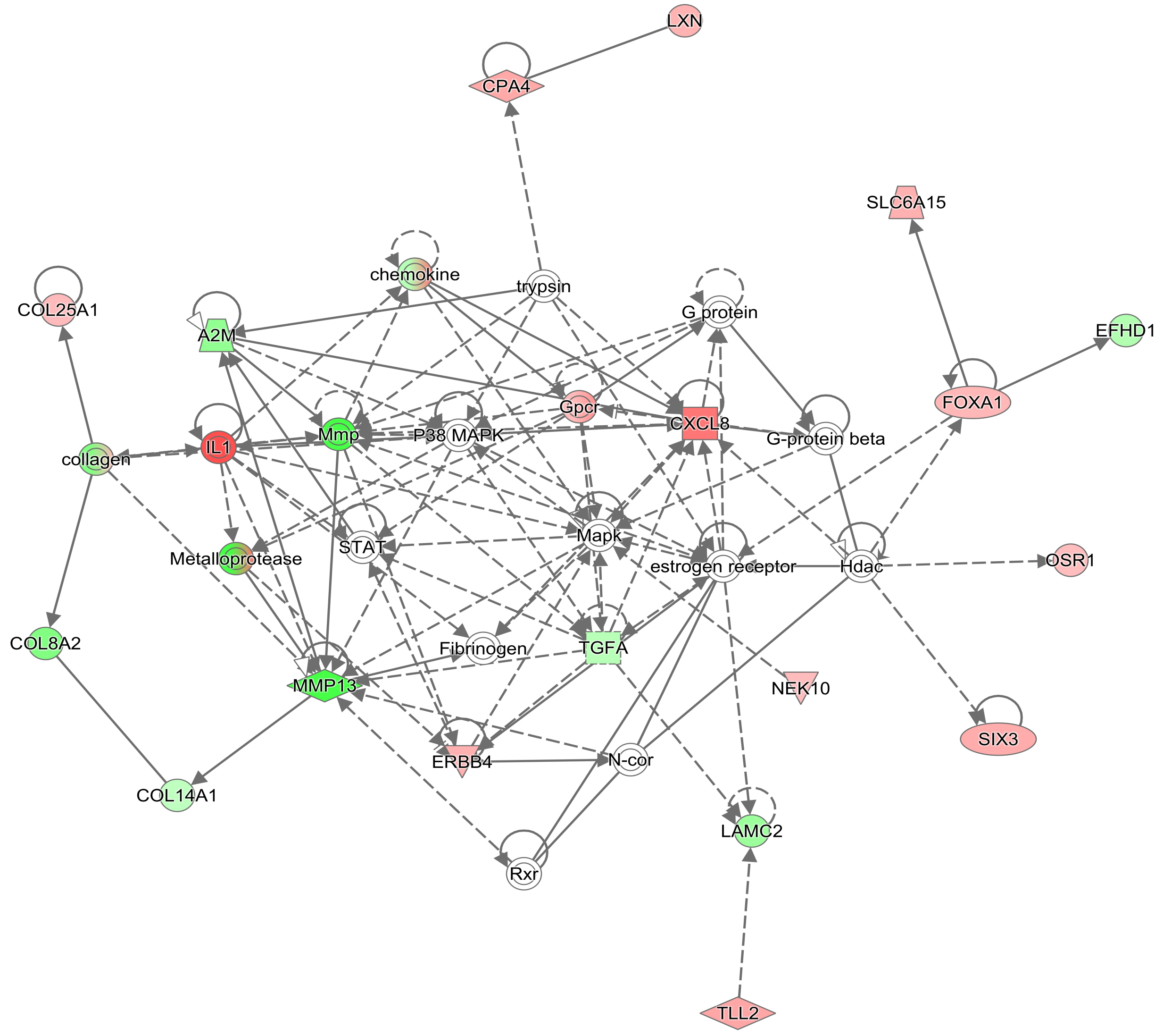

© 2000-2016 QIAGEN. All rights reserved.

S3.2. Network of genes significantly differentially expressed in untreated vs. 48h samples.

Network 1. Cancer, Connective Tissue Disorders, Organismal Injury and Abnormalities

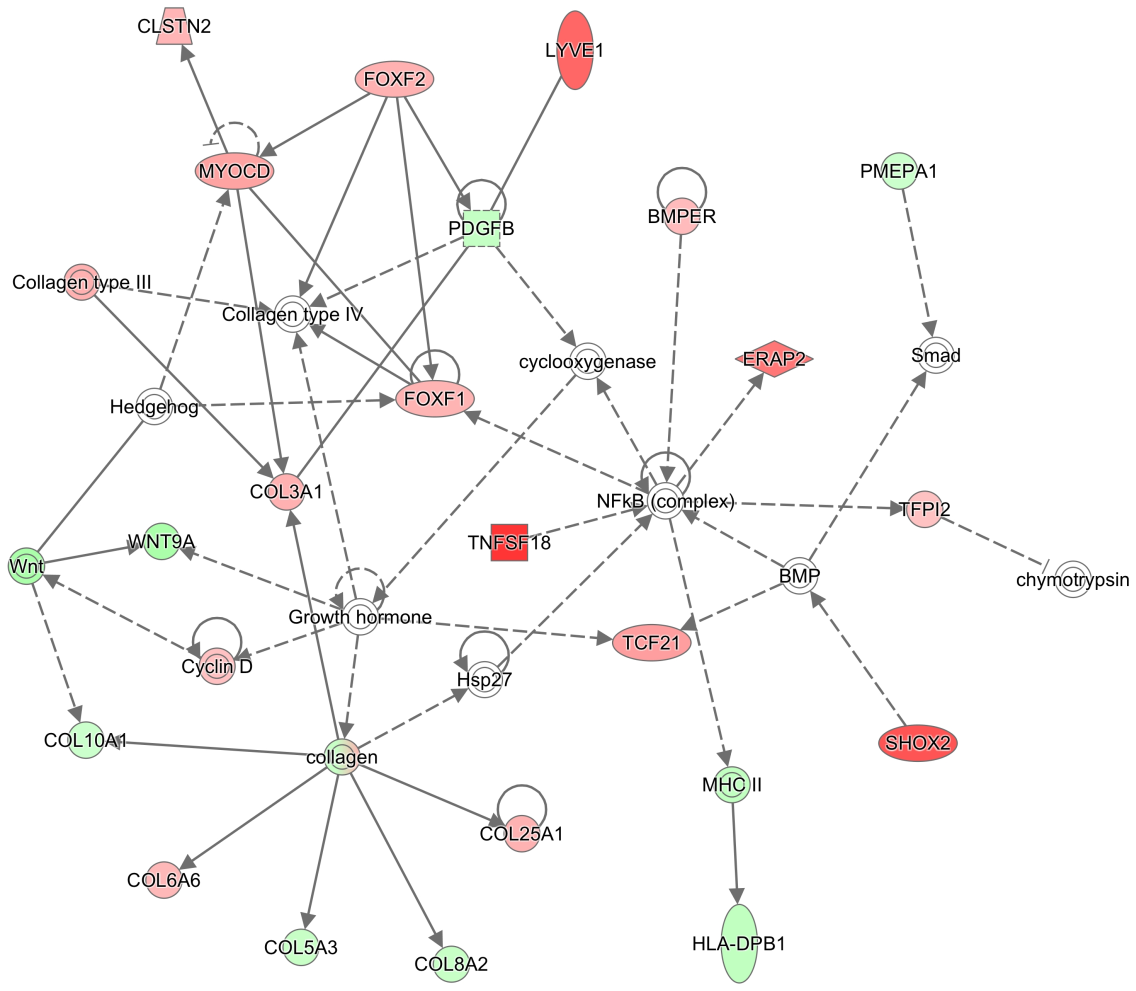

© 2000-2016 QIAGEN. All rights reserved.

Network 2. Neurological Disease, Skeletal and Muscular Disorders, Cancer

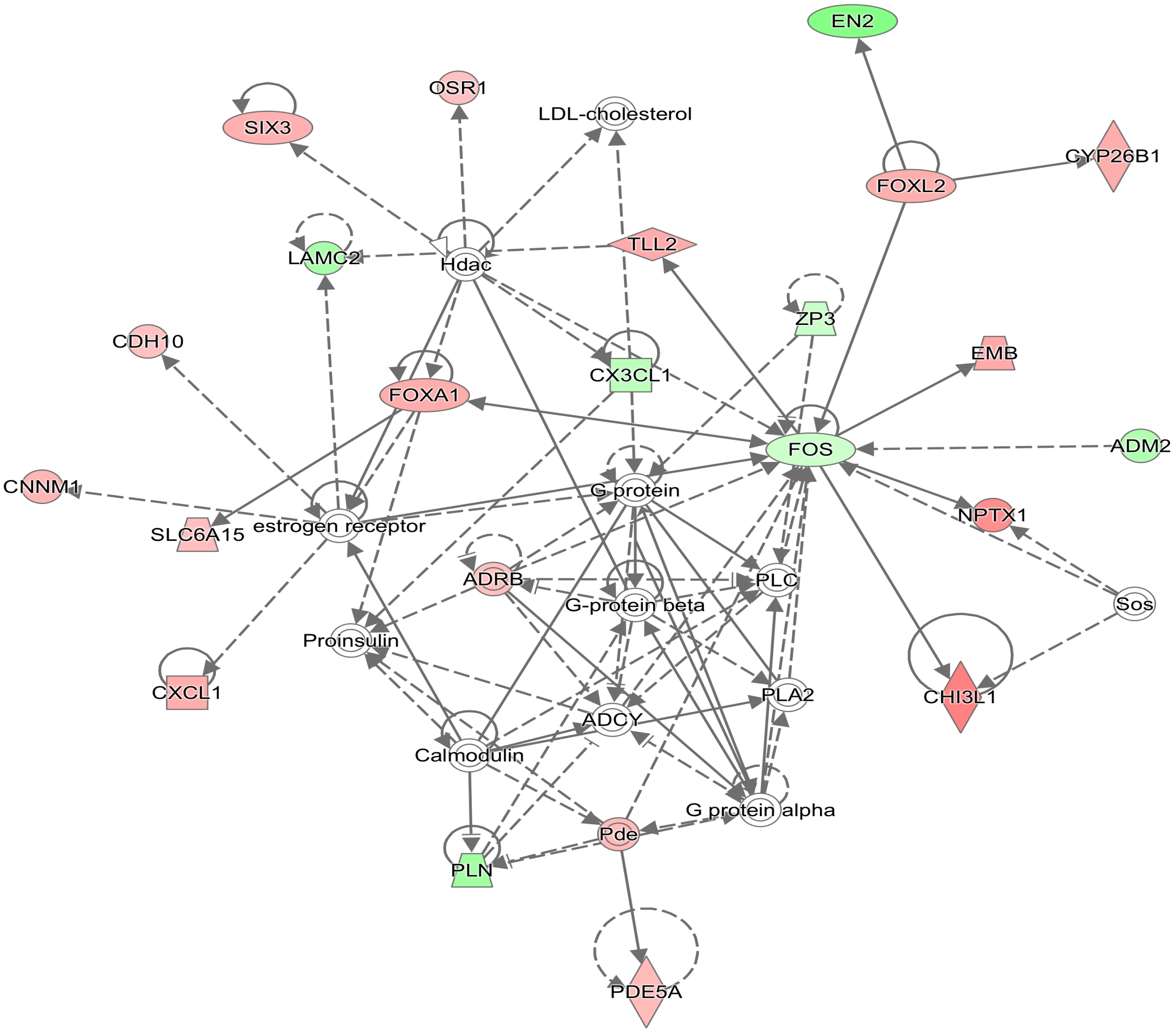

© 2000-2016 QIAGEN. All rights reserved.

Network 3. Cell-To-Cell Signaling and Interaction, Cellular Assembly and Organization, Cellular Movement

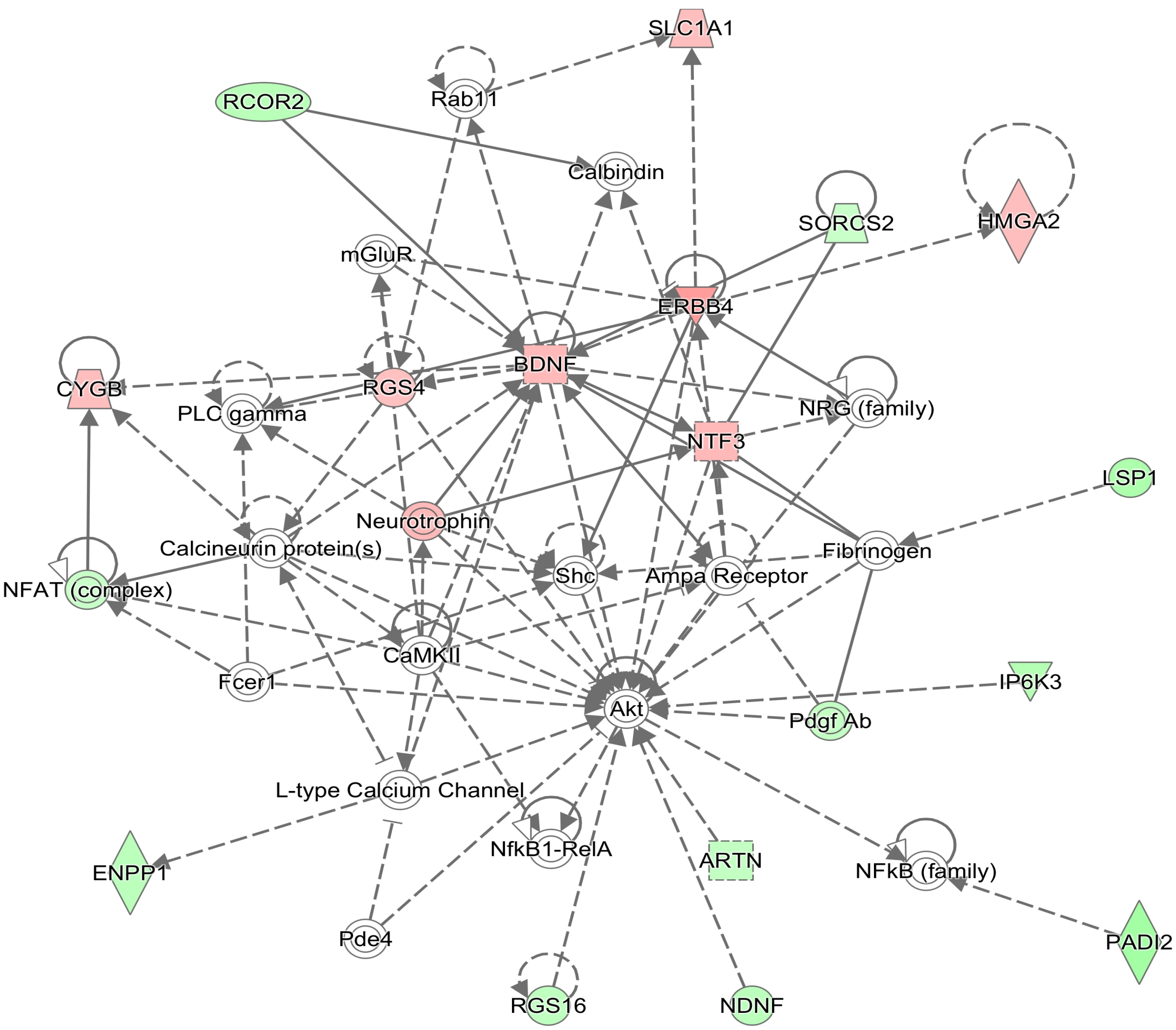

© 2000-2016 QIAGEN. All rights reserved.

Network 9. Cellular Movement, Hematological System Development and Function, Immune Cell Trafficking

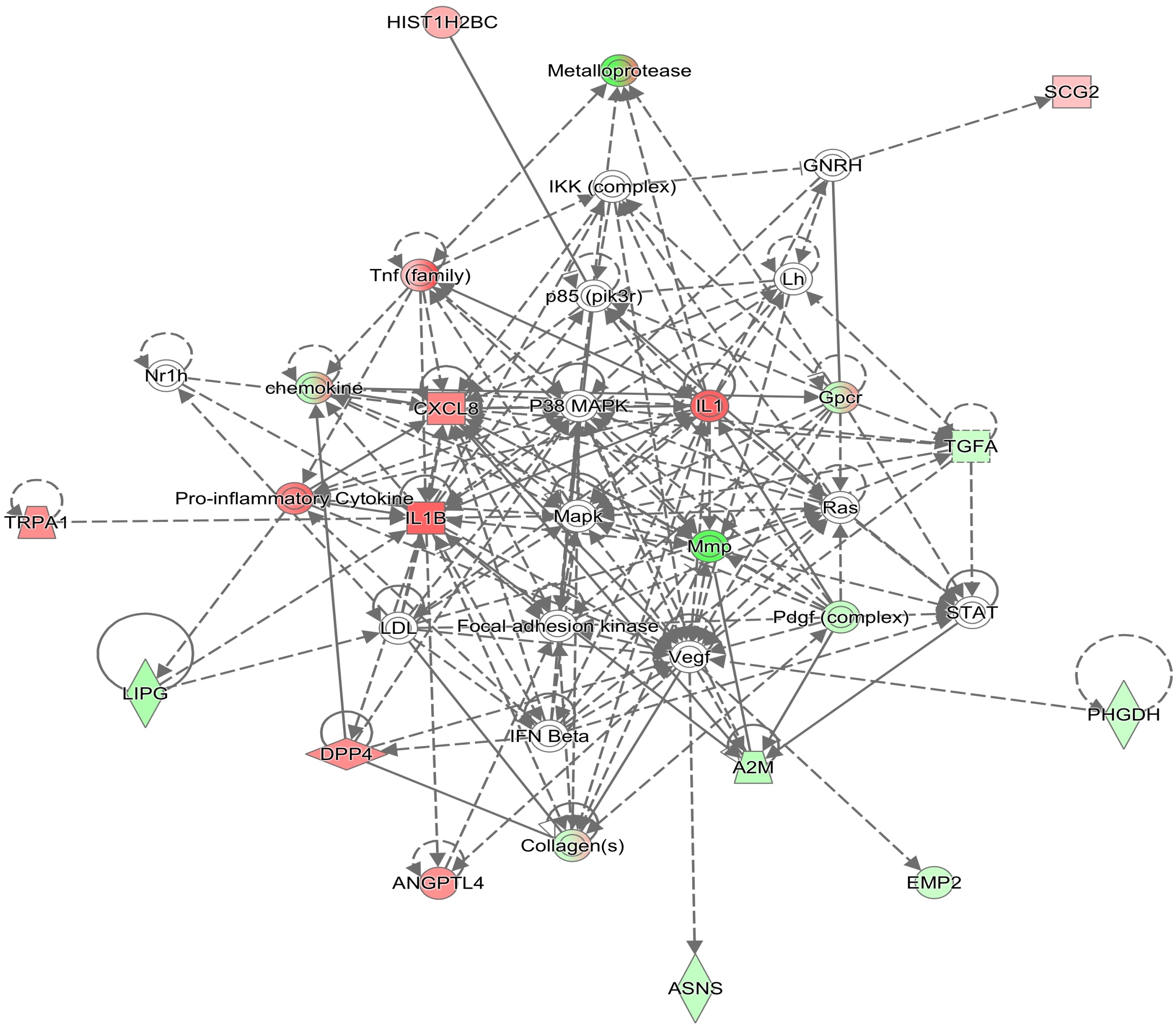

© 2000-2016 QIAGEN. All rights reserved.

Legends

Default Molecule Colors

- Dataset file genes. Non-Focus*
- KB genes - Not part of dataset file.
- Focus genes are colored according to user's preferences.
- Indicates shared nodes from 2 or more analyses.

*For dataset files that contain only identifiers (i.e. no expression values), the color gray identifies the Focus Genes from that dataset.

**Red**	User input molecule that is upregulated (ie. has a positive (+) expression value) and whose expression value meets the user defined cutoff.
**Green**	User input molecule that is downregulated (ie. has a negative (-) expression value) and whose expression value meets the user defined cutoff.
**Gray**	User input molecule. Neither up nor down-regulated or does not meet the user-defined cutoff.
**White**	Molecule that is not user specified, but incorporated into the network through relationships with other molecules.
**[Blue]**	For canonical pathways, molecules that are members of the network being examined are outlined in blue.

Molecule color intensity

The intensity of green and red molecule colors indicates the degree of down or upregulation, respectively. If normalized ratio, fold-change or log ratio/log fold-change is chosen as Expression Value type, a greater intensity of green represents a higher degree of downregulation, and a greater intensity of red represents a higher degree of upregulation. In contrast, for the expression value type p-value all Focus molecules are red by default; and a higher color intensity represents a lower (and thus more significant) p-value.

Molecule coloring can be customized by adjusting the Application Preferences.

Molecule Shapes

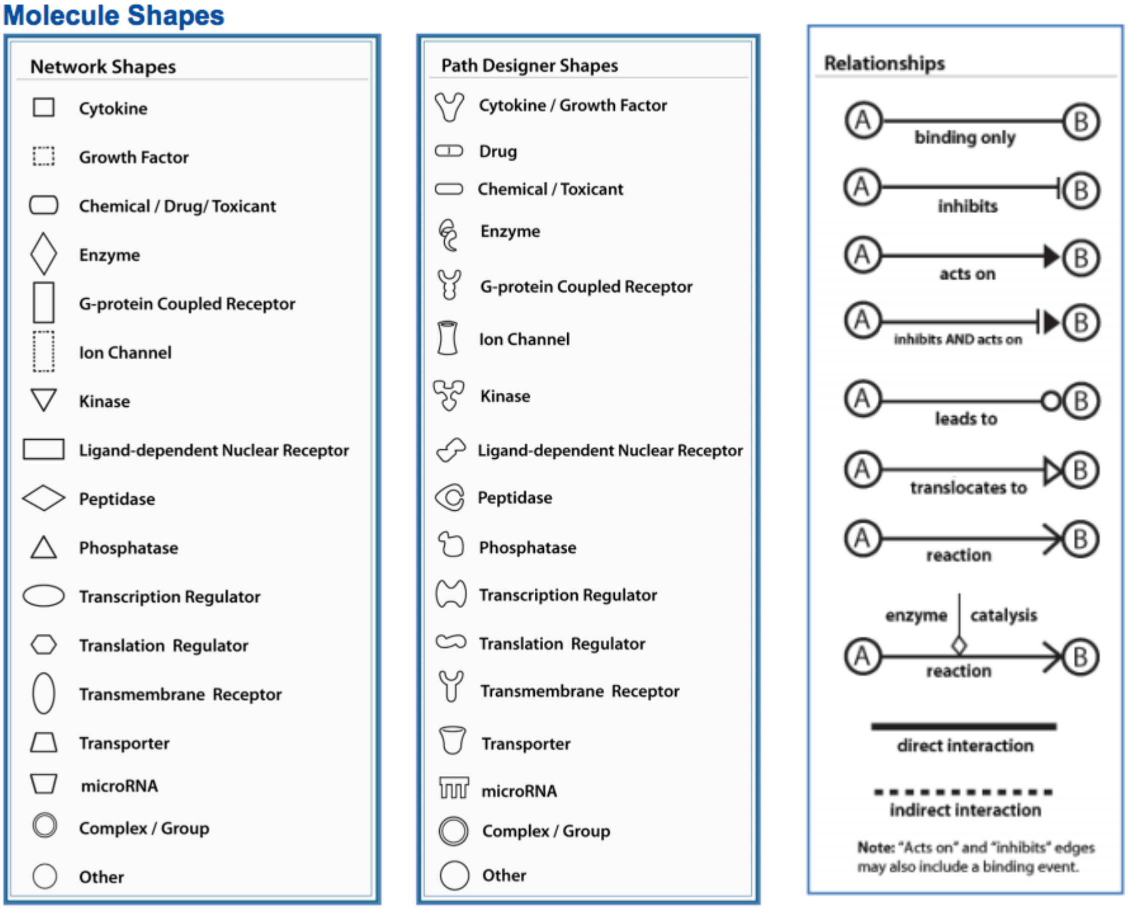

Relationship Labels

A Activation

B Binding

C Causes/Leads to

CC Chemical-Chemical interaction

CP Chemical-Protein interaction

E Expression (includes metabolism/ synthesis for chemicals)

EC Enzyme Catalysis

I Inhibition

L ProteoLysis (includes degradation for Chemicals)

LO Localization

M Biochemical Modification

MB Group/complex Membership

P Phosphorylation/Dephosphorylation

PD Protein-DNA binding

PP Protein-Protein binding

PR Protein-RNA binding

RB Regulation of Binding

RE Reaction

RR RNA-RNA Binding

T Transcription

TR Translocation
